# A prospective prognostic signature for pancreatic adenocarcinoma based on ubiquitination-related mRNA-lncRNA with experimental validation in vitro and vivo

**DOI:** 10.1007/s10142-023-01158-1

**Published:** 2023-08-04

**Authors:** Zhizhou Wang, Qihang Yuan, Xu Chen, Fei Luo, Xueying Shi, Fangyue Guo, Jie Ren, Shuang Li, Dong Shang

**Affiliations:** 1 Department of General Surgery, First Affiliated Hospital of Dalian Medical University, Dalian, Liaoning China; 2 Laboratory of Integrative Medicine, First Affiliated Hospital of Dalian Medical University, Dalian, Liaoning China; 3 Institute (College) of Integrative Medicine, Dalian Medical University, Dalian, Liaoning China; 4 Department of Oncology, First Affiliated Hospital of Dalian Medical University, Dalian, Liaoning China

**Keywords:** Pancreatic adenocarcinoma, Ubiquitination, Molecular clusters, Prognostic classification, Tumor immune microenvironment

## Abstract

Ubiquitination-related genes (URGs) exerted a crucial part in a variety of human disease disorders; however, their association with pancreatic adenocarcinoma (PAAD) had yet to be clearly described. We aimed to comprehensively characterize the contributions of URGs in PAAD through in silico analysis and experimental validation, and then identified a robust mRNA-lncRNA-based molecular prognostic panel for patients with PAAD using bulk RNA-sequencing and single-cell RNA-sequencing data. Initially, we collected the multi-omics data from TCGA platform to depict a comprehensive landscape of URGs in pan-cancer. Furthermore, we were accurate to PAAD for in-depth analysis. Significant differences of the activation of ubiquitination pathways and the expression of URGs were detected between normal and malignant cells. Unsupervised hierarchical clustering determined two PAAD subtypes with distinct clinical outcomes, ubiquitination pathway activities, immune microenvironment, and functional annotation characteristics. The expression profiles of ubiquitination-associated mRNAs and lncRNAs in the training and validation datasets were utilized to develop and verify a novel ubiquitination-related mRNA-lncRNA prognostic panel, which had a satisfied prediction efficiency. Our ubiquitination-associated model could function as an effective prognostic index and outperformed four other recognized panels in evaluating PAAD patients’ survival status. Tumor immune microenvironment, mutation burden, and chemotherapy response were intensively explored to demonstrate the underlying mechanism of prognostic difference according to our panel. Our findings also revealed that FTI-277, a farnesyltransferase inhibitor, had a better curative effect in high-risk patients, while MK-2206, an Akt allosteric inhibitor, had a superior therapeutic effect in low-risk patients. The real-time PCR results uncovered the RNA expression of AC005062.1 in all the three PAAD cell lines was elevated several thousandfold. In conclusion, our URGs-based classification panel could be triumphantly served as a prediction tool for survival evaluation in patients with PAAD, and the genes in this panel could be developed as a potential target in PAAD therapy.

**Supplementary Information:**

The online version contains supplementary material available at 10.1007/s10142-023-01158-1.

## Introduction

Pancreatic adenocarcinoma (PAAD), as a typical malignant tumor in digestive disease, occurs secretively and develops rapidly with poor therapeutic effects and prognosis. As the seventh primary death from cancer worldwide, PAAD accounts for approximately 459,000 new cases and 432,000 deaths (Ryan et al. 2014; Bray et al. 2018). Due to the fact that it is frequently detected at the terminal stage and is resistant to therapy, PAAD is a remarkably deadly disease with a lowest 5-year survival rate of roughly 9% in all cancers (Siegel et al. 2020). The effective strategies of both diagnostic program and prognostic evaluation are desperately needed for detecting tumors at an early stage and distinguishing risk stratification in patients with PAAD (Mizrahi et al. 2020). Consequently, a wide exploration of pathogenesis, biomarkers, early stratification, and prognostic evaluation has been investigated for the individual management of patients with PAAD. Up to the present, plentiful bioinformatic studies have revealed that microRNA, lncRNA, and other biomolecules can affect the progression, metastasis, prognosis, and immunotherapy of pancreatic cancer through regulating oxidative stress, immune response, and tumor cell proliferation (Liu et al. 2018; Wu et al. 2020a; Yu et al. 2021; Altan and Sahin 2022; Huang et al. 2023). Although these prognostic signatures of PAAD have been developed, most of them are accompanied by unsatisfied diagnostic values. More importantly, there is no accordant risk delamination for PAAD.

Ubiquitination, as a common and important process in cells, is one of the protein post-translational modification types, which plays an essential role in modulating substrate degradation, serving to regulate the functions of many proteins under various physiological and/or pathological conditions (Shmueli and Oren 2005; Popovic et al. 2014). The significant role of ubiquitination in cancer development and progression has been demonstrated in multiple reviews, which detailly comments the regulating mechanism of ubiquitination-related biomolecules on neoplastic diseases (Hoeller and Dikic 2009; Kirkin and Dikic 2011; Lipkowitz and Weissman 2011; Deng et al. 2020). There have been reports that ubiquitination-related regulators are discovered to evaluate the prognostic prediction in renal cell carcinoma and bladder cancers (Cai et al. 2021; Wu et al. 2021; Zhou et al. 2021). Additionally, studies in vivo and vitro display that ubiquitination-related regulators, such as COP9 signalosome complex subunit 6 (CSN6) and F-box protein 22 (FBXO22), participate in the progression of PAAD (Ma et al. 2020; Chen et al. 2021). Therefore, it is prospective to develop a PAAD risk stratification tool based on URGs.

In our study, we firstly depicted the landscape of ubiquitination-related mRNAs in pan-cancers, highlighting their significant contributions to multiple human cancers. The scRNA-seq data of PAAD samples also verified the close association of ubiquitination pathway and PAAD progression. Next, we especially focused our attention on PAAD. With the help of a series of bioinformatics analyses, we successfully established ubiquitination-related mRNA-lncRNA regulation network, and PAAD patients were stratified into two subtypes with distinct ubiquitination activities. We also established a novel ubiquitination-related prognostic signature (URPS) and verified its ability to predict the prognostic risk for PAAD. The real-time PCR experiments revealed the expression profiles of ubiquitination-associated mRNA and lncRNAs in URPS. Our research could provide new avenues for the clinical decision and prognostic evaluation in PAAD.

## Methods

### Pan-cancer analysis

The pan-cancer transcriptomes about the copy number variations (CNV), single-nucleotide variant (SNV), mRNA expression profiles, and clinical outcomes were acquired and integrated based on The Cancer Genome Atlas (TCGA) platform. The CNV, SNV, and expression of ubiquitination-related mRNAs in pan-cancers were summarized and shown with the help of the Perl language and TBtools. The ubiquitination score of tumor sample was computed to illuminate the varied function of signaling pathways impacted by ubiquitination among cancer types using single-sample gene set enrichment analysis (ssGSEA) (Fan et al. 2022). Samples that scored in the top 30% and the bottom 30% of ubiquitination were picked out as two representative groups for in-depth investigation about the discrepancies of pathway activities depending on gene set enrichment analysis (GSEA) (Guo et al. 2022).

### Single-cell RNA-seq data collection and bioinformatics analysis

The expression profiles and clinical information of 57,530 cells from 24 PAAD cases were provided by the CRA001160 dataset from the Genome Sequence Archive (GSA, https://ngdc.cncb.ac.cn/gsa/) for our analysis. The scRNA data was processed using Seurat in the R programme, and the percentage of mitochondria and rRNA was determined using “PercentageFeatureSet” function. The scRNA-seq data was standardized by “LogNormalize” technique. The top 2000 variable genes identified by FindVariableGenes tool were used to run the principal component analysis (PCA) (Pan et al. 2023). The t-distributed stochastic neighbor embedding (t-SNE) approach was used to do dimensionality reduction on the initial 50 PC. The CellCycleScoring tool was utilized to determine the cycle phase-specific alterations of cells in distinct samples. The CellCycleScoring program in R scored each cell based on the expression of G2/M and S phase markers, which were applied to determine cell cycle phase (G2/M, S, or G1 phase).

Molecular Signatures Database (MsigDB, https://www.gsea-msigdb.org/gsea/msigdb/) was the source for the Hallmark gene collection. From the MsigDB of Gene Set Enrichment Analysis (GSEA, http://www.gsea-msigdb.org/gsea/index.jsp), 79 ubiquitination-related genes (URGs) were retrieved. Normal and malignant cells from 24 PAAD samples in CRA001160 were analyzed using ssGSEA to identify enrichment pathways.

### Bulk RNA-seq data collection and processing

The transcriptome profiling and survival data of normal and PAAD samples were acquired from Genotype-Tissue Expression project (GTEx) and International Cancer Genome Consortium (ICGC) databases. In total, we obtained 178 PAAD and 4 normal pancreas samples from TCGA cohort, 167 normal pancreas samples from GTEx cohort, and 84 PAAD samples from ICGC cohort. After obtaining intersected molecules in all the cohorts, batching normalization was conducted for the following analysis. Among them, 64 URGs with expression data were collected and 15 URGs were excluded due to incomplete expression data. Additionally, in order to get lncRNAs from genes that overlapped, the human gene transfer format (gtf) file was downloaded from Ensembl website (http://www.ensembl.org/).

### Identification of differentially expressed ubiquitination-related mRNAs and lncRNAs with prognostic values

Pearson correlation coefficients between 64 ubiquitination-related mRNAs and all lncRNAs were determined using R’s in-built “cor.test” function. We screened ubiquitination-related lncRNAs in terms of the correlation coefficients and *p* values (|correlation coefficients| > 0.4 and *p* values < 0.001). One hundred seventy-eight PAAD and 4 normal pancreas samples from TCGA dataset and 167 normal pancreas samples from GTEx dataset were combined (i.e., 178 PAAD and 171 normal samples). Furthermore, the expression matrix of ubiquitination-associated mRNAs and lncRNAs that coordinated with clinical information was integrated for further analysis in PAAD. The “limma” package in R was employed to obtain the differentially expressed ubiquitination-related mRNAs and lncRNAs between normal and PAAD samples depending on the false discovery rate (FDR) and |log2(fold change)| (FDR < 0.01 and |log2(fold change)| > 1). In addition, in TCGA cohort, ubiquitination-related mRNAs and lncRNAs with prognostic profiles were picked out using univariate Cox analysis modulated by Benjamini and Hochberg (BH) method (FDR < 0.05). Ultimately, ubiquitination-related mRNAs and lncRNAs with both differential expression and prognostic values were retained for subsequent consensus clustering and panel development.

### Consensus clustering analysis identifies ubiquitination-related mRNA-lncRNA-based molecular clusters

Using the “ConsensusClusterPlus” package, 178 PAAD specimens from TCGA cohort were segmented into two clusters (i.e., C1 and C2) based on the above ubiquitination-related mRNAs and lncRNAs with both differential expression and prognostic values. The prognostic discrepancies of the clusters were estimated using survival analysis with the help of Kaplan-Meier method. We employed four indicators to estimate the survival analysis, including overall survival (OS), progression-free interval (PFI), disease-specific survival (DSS), and disease-free interval (DFI). The ubiquitination pathway activities were determined by the ubiquitination pathway scores of patients with PAAD, which were computed using “GSVA” package in R. Then, we compared the discrepancy of the ubiquitination scores between distinct ubiquitination-based clusters using the “wilcox.test” function in R. We also investigated the discrepancy of clinicopathological traits between these two clusters, including age, gender, grade, stage, and survival status. To discover the functional characteristics of each cluster, the specific molecules of them were then determined. The definition of specific molecules was as follows: the molecules that were significantly upregulated or downregulated in cluster 2 contrasted to cluster 1 were rated as the specific molecules. Cytoscape plug-in ClueGO, CluePedia, and yFiles Layout Algorithms were employed to explore the potential gene ontology terms enriched by these specific molecules for further investigation of the underlying mechanism of prognostic heterogeneity.

### Cluster-based exploration of the discrepancy of tumor immune microenvironment (TIME)

The immune microenvironment of each PAAD sample was assessed by means of the “estimate” package in R. After that, the “ggpubr” package in R was employed to visualize the result of the immune microenvironment. The infiltrating compositions of 22 immune cells were analyzed using CIBERSORT method in PAAD samples. The “wilcox.test” function in R was used to further examine the disparity in immune cell infiltration (ICI) and immunological checkpoint gene (ICG) expression between the two clusters.

### Identification and verification of an ubiquitination-related mRNA-lncRNA-based prognostic panel

All PAAD samples (178 PAAD samples obtained from TCGA dataset; 84 PAAD samples obtained from ICGC dataset) were included for the subsequent research. There were three samples (1 sample from TCGA dataset; 2 samples from ICGC dataset) excluded due to lack of survival time. In total, 177 PAAD samples from TCGA dataset and 82 PAAD samples from ICGC dataset were screened for subsequent research. Half of 177 PAAD samples obtained from TCGA dataset were randomly distributed in the train cohort. All 177 PAAD samples obtained from TCGA dataset and 82 PAAD samples from ICGC dataset were allocated to test1 and test2 cohorts, respectively. Notably, test1 cohort was rated as internal validation group, while test2 cohort was set as external validation group.

In the train cohort, the collinear elimination of the 79 variables and over-fitting prevention of the established model were accomplished with the help of least absolute shrinkage and selection operator (LASSO) regression (Li et al. 2022). Then, multivariate Cox proportional hazards regression analysis was further used for the construction of a novel URPS. In the train and test cohorts, PAAD specimens were divided into low-risk and high-risk subgroups using “predict” function in R, with the critical value determined by the median risk score.

The following analyses were performed to construct the URPS and validate its robustness: (1) Survival analyses including OS, PFI, DSS, and DFI were used for the assessment of predictive ability of URPS with the help of Kaplan-Meier method in the train and test1 cohorts. (2) The receiver operating characteristic (ROC) curve was employed to verify the diagnostic values of URPS at differential years among distinct clinical features using “survivalROC” package in R. (3) Four additional prognostic signs of PAAD were analyzed, and their predictive efficacy was compared to that of our URPS (a ferroptosis-related model established by Yu et al (Yu et al. 2021), a RNA-binding protein-related prognostic model established by Wen et al (Wen et al. 2021), a metastasis-related prognostic model established by Wu et al (Wu et al. 2020b), and an immune-related model established by Wu et al (Wu et al. 2020a) under the help of “survival,” “tidyverse,” and “timeROC” packages in R in the train and test1 cohorts. Because of the insufficient clinical information in the ICGC database, only the survival analysis of OS was performed, and the diagnostic values of URPS at 0.5 year, 1 year, 2 years, and 3 years were verified without the comparison of different clinical features in the test2 cohort. Finally, URPS-based risk scores together with other clinicopathological indexes were incorporated for the investigation of independent prognostic indicators by utilizing variate Cox regression analyses in PAAD. Because of the deficiency of clinical data in ICGC dataset, variate Cox regression analyses were only conducted depending on the 177 PAAD samples from the TCGA dataset.

### URPS-based pathway annotation

Recently, it has been confirmed that the genesis and progression of cancers are influenced by abnormal signaling pathways (Pan et al. 2023). One hundred eighty-six classical KEGG signaling pathways were collected from the MsigDB database. The pathway activities of patients with PAAD were computed using ssGSEA in the training, testing1, and testing2 cohorts, and “wilcox.test” function in R was then employed to compare the pathway activities in low-risk and high-risk populations. The differential abundance of pathways with statistical discrepancy between low-risk and high-risk populations was visualized via the “pheatmap” package in R. The “ggcor” and “ggplot” packages in R were also implemented to compute and draw the correlation between risk score and pathway activities. Following these, the venn plot was utilized to identify the common pathways among the training, testing1, and testing2 cohorts.

### URPS-based characterization of the TIME

Numerous immune deconvolution techniques were modified to evaluate the discrepancies of the infiltrating abundances of immune cells in low-risk and high-risk populations, which included TIMER, QUANTISEQ, CIBERSORT, MCPCOUNTER, and XCELL. A heatmap was produced to depict the infiltrating overview about immune cells. Furthermore, we also explored the discrepancies of expression levels of ICGs between low-risk and high-risk populations. Notably, only the results that had statistical significance were displayed.

In 2018, there was an article that published in the “Immunity” journal by Thorsson, which detected six immune subtypes of more than 10,000 tumors among 33 cancer types from TCGA dataset (Thorsson et al. 2018). Thus, we then evaluated the differences in immunological subtypes between low-risk and high-risk subgroups using the techniques described in the study.

### URPS-based characterization of the tumor mutation burden (TMB)

It is greatly evident that high TMB suggests a satisfied response to immunotherapy (Sui et al. 2023). In consideration of the clinical significance of TMB, the information of tumor mutation in PAAD patients from TCGA dataset was downloaded for deep analysis. The quantity of non-synonymous mutations was enumerated using Perl. The entire quantity of somatic gene coding errors, base substitutions, gene interposition, or removal errors tested per million bases was defined as TMB.

The TMB scores in low-risk and high-risk populations were analyzed using the “wilcox” function in R. Spearman correlation analysis was performed to explored the association between risk scores and TMB scores. Then, we used “maftools” R programme to isolate the genes responsible for PAAD, and we extended our diagram to include the current standing of the top 20 genes in two distinct categories. We also explored the clinical outcomes in PAAD patients with distinct TMB and risk scores using K-M log rank test.

### Targeted drug sensitivity prediction

Drug sensitivity was predicted for each PAAD patient using the “pRRophetic” R package, and the “wilcox.test” R function was used to evaluate the possibly sensitive medications in low-risk and high-risk populations. Targeted medications were deemed trustworthy when they showed statistical significance in all three cohorts (train, test1, and test2). Notably, medication sensitivity increases as the IC50 value decreases.

### Cell lines and culture

HPDE6-C7 cell line was chalked up from American Type Culture Collection (ATCC, USA). CF-PAC1 and Panc-1 cell lines were amicably supported by Procell Life Science & Technology Co., Ltd. BxPC-3 cell line was obtained from KeyGEN BioTECH (Jiangsu province, China). The cell lines of HPDE6-C7, BxPC-3, and Panc-1 were cultured in DMEM (Gibco, USA), whereas CF-PAC1 cell line was cultured in IMEM (Procell, China). All of medium were replenished with 10% fetal bovine serum (Gibco, USA).

### Clinical human samples

Paired cancerous and para-cancerous pancreatic tissues were collected from eight patients with PAAD and rapidly froze in liquid nitrogen for RNA extraction. Each of patient signed the informed consent form supplied by the First Affiliated Hospital of Dalian Medical University.

### Quantitative real-time PCR (qRT-PCR)

Total RNAs of cell lines and tissues of human pancreatic cancer were extracted using RNAex Pro Reagent (Accurate Biotechnology). To avoid RNA degradation, the RNA extraction of human pancreatic tissues was performed using liquid nitrogen grinding. The cDNAs were prepared using Evo M-MLV RT Kit with gDNA Clean (Accurate Biotechnology). The qRT-PCR technology was implemented using SYBR^®^ Green Premix Pro Taq HS qPCR Kit (Accurate Biotechnology, Shanghai, China). GAPDH was used as the control gene. The ^ΔΔ^Ct method was utilized to quantitate the gene expression. All of primer sequences employed in this research were as follows: for human UBE2C, 5′-GACCTGAGGTATAAGCTCTCGC-3′ (forward), 5′-TTACCCTGGGTGTCCACGTT-3′ (reverse); for human DANCR, 5′-GCGCCACTATGTAGCGGGTT-3′ (forward), 5′-TCAATGGCTTGTGCCTGTAGTT-3′ (reverse); for human AL139147.1, 5′-GCAGCCTCTACCAATGTGATG-3′ (forward), 5′-GGACAGTTTTCGTCATTCCCG-3′ (reverse); for human AC092171.2, 5′-GGTCATCGAAAGGCAGGTGA-3′ (forward), 5′-TTCGCCACCTTCTGAGCATT-3′ (reverse); for human AC005062.1, 5′-TTCTCTCGACTGAGCCAACACA-3′ (forward), 5′-GAGAGACAGAAAGCGGAGTCA-3′ (reverse); for human BX293535.1, 5′-ACTTCTGAGCCAGACTGCTTG-3′ (forward), 5′-AGTGAGTACATTCAAACCAGAACT-3′ (reverse); for human AC009065.5, 5′-TGAACCTCTGTTGTCTGTGGA-3′ (forward), 5′-GGAGCCTTTCTGCTCCTACAA-3′ (reverse); for human AP005233.2, 5′-CCAAAGAACCAAGAGCTGCA-3′ (forward), 5′-CAAACCCACAGACCCTCTCT-3′ (reverse); for human AC005261.1, 5′-GCCTGTTCAAGTCCCAACCT-3′ (forward), 5′-GGCCTCAATCCCTGACCTTT-3′ (reverse); for human GAPDH, 5′-GGTCTCCTCTGACTTCAACA-3′ (forward), 5′-GTGAGGGTCTCTCTCTTCCT-3′ (reverse). The above primers were purchased from GenePharma (Suzhou, China).

### Statistical analysis

The statistical analyses of quantitative data were performed using the Student’s *t*-test and the Wilcoxon rank-sum test. The normally distributed variables were analyzed by the Student’s *t*-test, whereas the nonnormally distributed variables were analyzed by the Wilcoxon rank-sum test. Kruskal-Wallis test and one-way ANOVA test were used to compare more than two groups as nonparametric and parametric methods, respectively. The R (version 4.1.1) and Perl languages were used to conduct all statistical analyses in this work (*p < 0.05, **p < 0.01, ***p < 0.001).

## Results

### Pan-cancer overview of the ubiquitination-related regulators

**Fig. 1:**
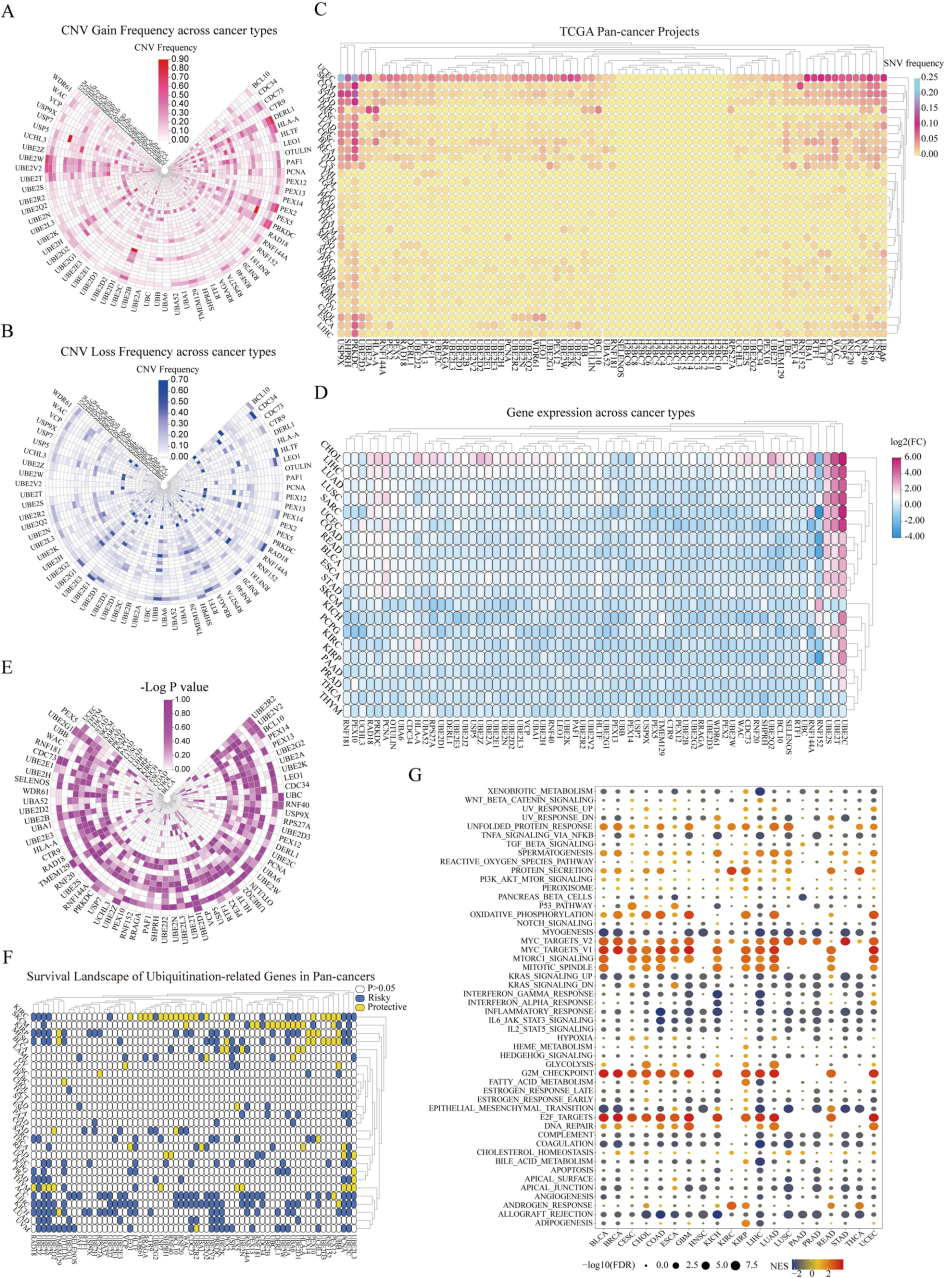
Pan-cancer overview of the ubiquitination-related genes. **A**, **B** CNV gain frequency and CNV loss frequency information among cancer types. The red color represents the gain frequency, whereas the blue color represents the loss frequency in the ubiquitination-related genes from pan-cancers. **C** SNV data among cancer types. The mutant frequencies of ubiquitination-associated genes in pan-cancers (red color to blue color represents low to high). **D**, **E** Differential expression traits of the ubiquitination-related genes in pan-cancers and their corresponding para-cancerous tissues (**D** log2FC values, **E** *p* values). **F** Prognostic contributions of the ubiquitination-related genes in pan-cancers. Genes (*p* > 0.05) were presented by white color, and the risky and protective genes were presented by blue and yellow colors, respectively. **G** Enrichment analysis for typical cancer-associated pathways between distinct URG-score crowds (NES, normalized enrichment score)

Even though a large number of ubiquitination-related regulators have been investigated in various cancers (Sun et al. 2020), the mutations of these genes are not well summarized. Consequently, we investigated the mutations of ubiquitination-related regulators in pan-cancers. The high-throughput CNV and SNV data gathered from TCGA database were analyzed and visualized as heatmaps. The result of CNV gain frequency showed that URGs had higher frequencies of gain mutations in kidney chromophobe (KICH), adrenocortical carcinoma (ACC), uterine carcinosarcoma (UCS), ovarian serous cystadenocarcinoma (OV), sarcoma (SARC), and rectal adenocarcinoma (READ), whereas derlin-1 (DERL1), ubiquitin-conjugating enzyme E2W (UBE2W), peroxisomal E3 ubiquitin ligase peroxin 2 (PEX2), ubiquitin-conjugating enzyme E2C (UBE2C), PRKDC, and ubiquitin-conjugating enzyme E2V2 (UBE2V2) had higher frequencies of gain mutations across various cancer types (Fig. 1A). The result of CNV loss frequency displayed that ubiquitination-related regulators had higher frequencies of loss mutations in KICH, OV, UCS, ACC, READ, and SARC, while ubiquitin-conjugating enzyme E2G1 (UBE2G1), UBB, peroxisomal E3 ubiquitin ligase peroxin 14 (PEX14), SNF2 histone linker PHD RING helicase (SHPRH), RING finger 152 (RNF152), and Ras-related GTP-binding A (RRAGA) had higher frequencies of loss mutations across various cancer types (Fig. 1B). In addition to this, the heatmap of SNV data showed higher mutation frequencies of ubiquitination-related regulators in uterine corpus endometrial carcinoma (UCEC), colon adenocarcinoma (COAD), skin cutaneous melanoma (SKCM), and stomach adenocarcinoma (STAD). Notably, protein kinase, DNA-activated, catalytic subunit (PRKDC), and ubiquitin-specific protease 9X (USP9X) had higher mutation frequencies in multiple cancers, mainly including UCEC, SKCM, COAD, and STAD (Fig. 1C).

To investigate the mRNA expression of ubiquitination-related regulators in various cancers, pan-cancer analysis was carried out with the help of gene expression data from TCGA database; then, the result was presented as the heatmap. The gene expressions of most ubiquitination-related regulators were low, while the gene expressions of UBE2C and ubiquitin-conjugating enzyme E2T (UBE2T) were extremely high in multiple types of cancer (Fig. 1D). To clearly display the gene expression changes, the negative log *p* value of each gene was utilized to create a new heatmap, which showed a comparatively high-degree variations of ubiquitination-related regulators in LIHC (liver hepatocellular carcinoma), KIRC, and LUSC (lung squamous cell carcinoma) (Fig. 1E). UBE2C and UBE2T were greatly changed in various cancers (Fig. 1E). Next, we integrated the gene expression and clinical survival time in TCGA dataset to intensively investigate the prognostic performances of the above ubiquitination-related regulators in pan-cancers. The univariate cox regression analysis identified ubiquitination-related regulators as risky (HR > 1 and *p* < 0.05) or protective (HR < 1 and *p* < 0.05) factors, and most of those were valuable in PAAD prognosis (Fig. 1F). Following this, we investigated how these ubiquitination-related regulators modulated the cancer-related signaling pathways in various cancer types by the GSEA. The result is presented as the heatmap in Fig. 1G, which revealed that ubiquitination scores were positively correlated to MYC_TARGETS_V1 and V2, MTORC1_SIGNALING, G2M_CHECKPOINT, and E2F_TARGETS, whereas negatively correlated to MYOGENESIS, INFLAMMATORY_RESPONSE, and ALLOGRAFT_REJECTION in multiple cancers.

### The scRNA-seq data unveils the close association between ubiquitination and PAAD

**Fig. 2:**
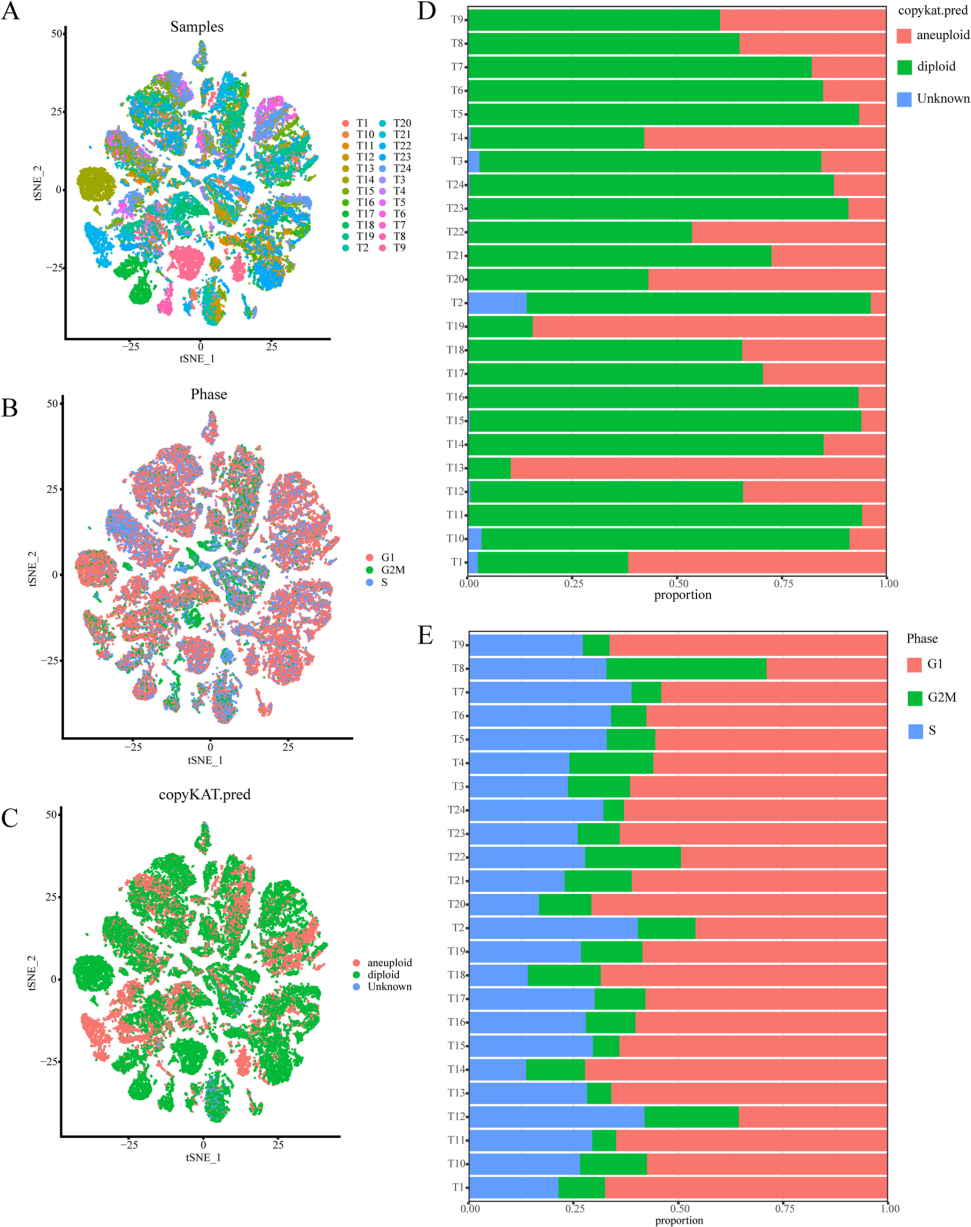
Characterization of cell types and cell cycles in pancreatic cancer. **A** The t-SNE algorithm identified cell clusters of pancreatic cancer. **B** Annotation of cells during various phases of the cell cycle in pancreatic cancer. **C** Malignant and nonmalignant cells in PAAD samples were depicted by various colors in t-SNE plots. **D** In each PAAD sample, the percentage of malignant to benign cells. **E** Proportion of cells in the G1, G2/M, and S phases in each PAAD sample

Next, we utilized the scRNA data to explore the correlation between ubiquitination and PAAD. The 24,005 genes from 57,530 cells were gathered for examination in line with the quality control criterion of scRNA-seq data (Supplementary Figure 1A). Even though there is a substantial link between UMI and mRNA, UMI/mRNA had no relationship with mitochondrial gene content (Supplementary Figure 1B). PCA was applied to assess the available dimensions, which showed that PAAD cells were not significantly distinct (Supplementary Figure 1C). Fifty of the most significant PCs were selected for in-depth analysis (Supplementary Figure 1C). The t-SNE was used to visualize the unsupervised clustering of cells, displaying the distribution of cells in each sample using distinct colors (Fig. 2A). In light of the consensus that proliferation is a primary hallmark for tumor cells, we labeled human PAAD cells in various phases of cell cycle (Fig. 2B). In addition, we annotated the distribution of nonmalignant and malignant cells in PAAD (Fig. 2C). In the majority of samples, malignant cells had much less percentage than normal cells (Fig. 2D). The majority of cells in PAAD samples were found to be in G1 phase, while only a tiny proportion of the cells located in the G2/M phase (Fig. 2E).

**Fig. 3:**
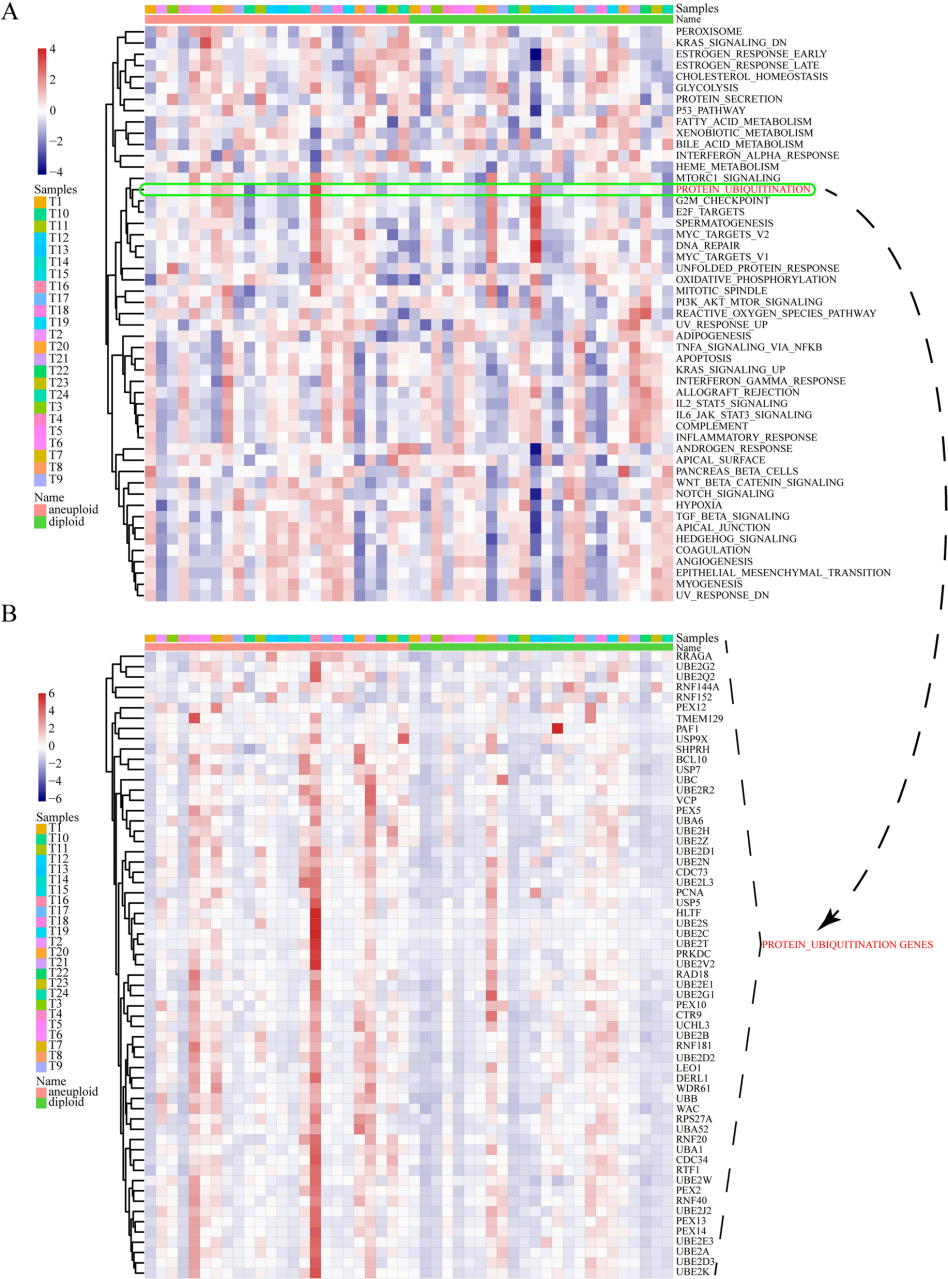
Identification of ubiquitination characteristics of pancreatic cancer in the scRNA-seq level. **A** In PAAD, normal and cancerous cells activated different biochemical pathways. **B** Difference in expression of URGs between malignant and nonmalignant cells in PAAD

Apart from that, we also compared the expression difference of several tumor-associated regulatory pathways between malignant and nonmalignant cells in PAAD. Other from nonmalignant cells, a variety of classical carcinogenesis pathways were comparatively active in malignant cells (Fig. 3A). It should be noted that protein ubiquitination was among these carcinogenesis pathways (Fig. 3A). More importantly, most of URGs showed enhanced expression levels in malignant cells contrasted to normal cells (Fig. 3B). The enrichment score of each pathway and the expression of each URG are also demonstrated in Supplementary Figure 2. Therefore, ubiquitination could be crucial for cellular malignization. Construction of ubiquitination-based molecular classification and risk stratification is promising for precise prediction of PAAD prognosis.

### Identification of ubiquitination-related mRNAs and lncRNAs with traits of both differential expression and prognostic values

To date, besides functional mRNAs, numerous lncRNAs have been characterized as functional molecules that are associated with different types of cancers by cancer transcriptome analysis (Li et al. 2020). It has been reported that lncRNAs are explored for the early detection and prognostic evaluation in PAAD (Namkung et al. 2016; Eid et al. 2021). Hence, the role of ubiquitination-related mRNAs and lncRNAs in the prognosis of PAAD was further investigated. Pearson correlation analysis determined 396 ubiquitination-related lncRNAs in PAAD (Supplementary Figure 3A). The analysis of 178 PAAD and 171 normal pancreas samples identified the expression profiles of 64 ubiquitination-associated mRNAs and 396 ubiquitination-associated lncRNAs. Then, a total of 263 differentially expressed ubiquitination-associated mRNAs and lncRNAs between PAAD and normal pancreas tissues were screened by Wilcoxon test in R language (Supplementary Table 1). Following that, 136 ubiquitination-related mRNAs and lncRNAs with prognostic values were screened in PAAD samples by univariate Cox regression analysis (Supplementary Table 2). Seventy-nine ubiquitination-related mRNAs and lncRNAs with traits of both differential expression and prognostic values were preserved after taking an intersection (Supplementary Figure 3B). The heatmap showed the distribution of the above 79 ubiquitination-related RNAs in PAAD and normal pancreas samples (Supplementary Figure 3C). Univariate Cox regression analysis showed that these 79 ubiquitination-related genes could serve as the potential prognostic markers, being the protective or risky factors in PAAD (Supplementary Figure 3D).

### Consensus clustering analysis identifies ubiquitination-related mRNA-lncRNA-based molecular clusters

**Fig. 4:**
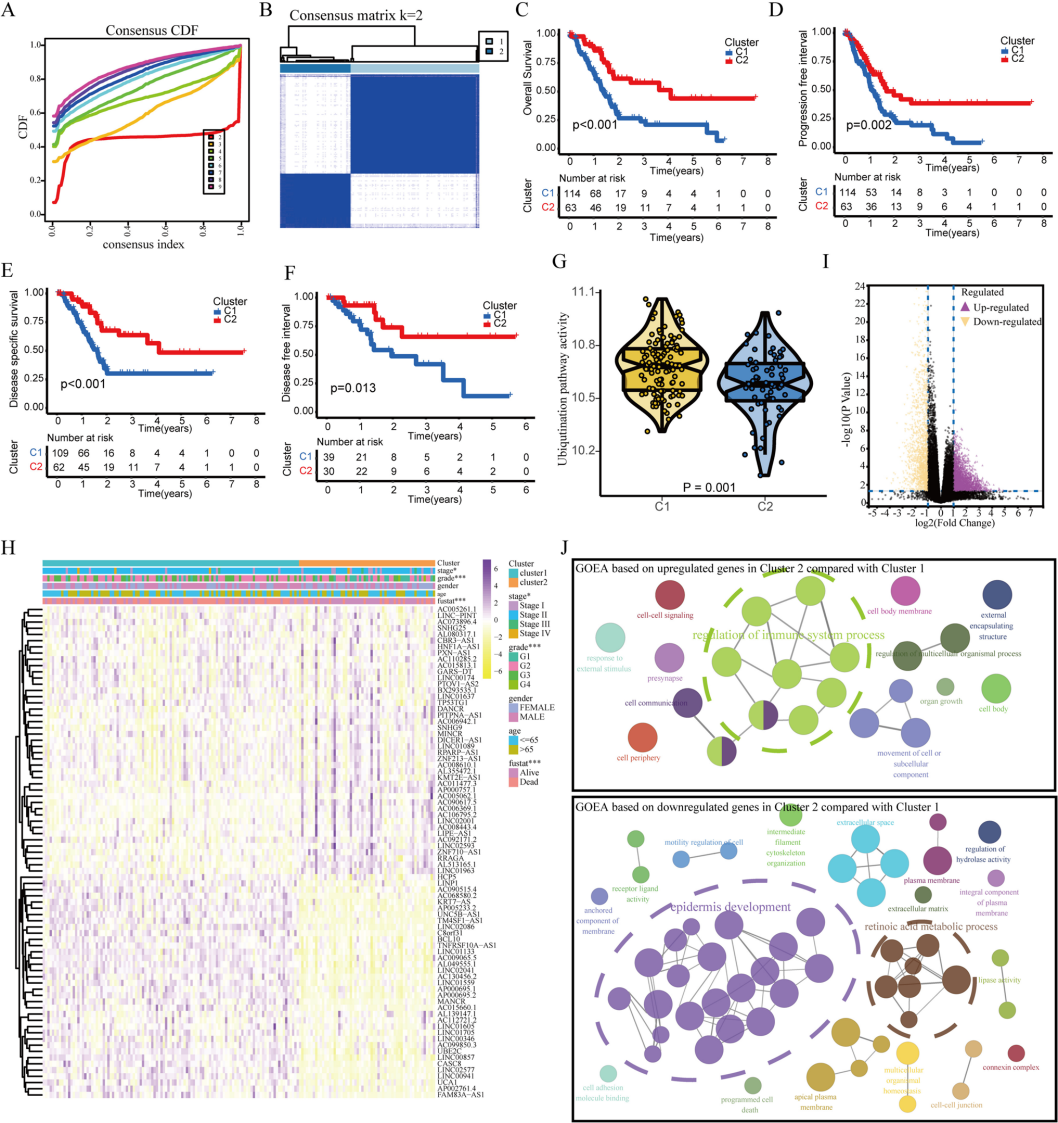
Identification of ubiquitination-related clusters in PAAD using consensus clustering analysis. **A** The consensus CDF curve when *k* is between 2 and 9. **B** The correlation between two ubiquitination-related clusters when *k* value is 2. **C–F** Comparison of OS, PFI, DSS, and DFI between two ubiquitination-related clusters. **G** Relationship of ubiquitination pathway activities and ubiquitination-related clusters. **H** Heatmap shows the correlation between ubiquitination-related clusters and clinicopathologic traits. **I** Volcano plot displayed 2689 upregulated and 1656 downregulated genes in cluster 2 compared with cluster 1. **J** GOEA was generated based on 2689 upregulated and 1656 downregulated genes with the Cytoscape plug-in ClueGO, CluePedia, and yFiles Layout Algorithms. The different colors of node represented the different GO results of 2689 upregulated and 1656 downregulated genes. The size of the node represented *p* value, and the *p* values of all nodes are < 0.05

To identify ubiquitination-based molecular clusters of PAAD, the above 79 ubiquitination-related mRNAs and lncRNAs with traits of both differential expression and prognostic values were utilized to perform consensus clustering analysis. In terms of cumulative distribution function (CDF) curves and Delta area, the quantity of subtypes was determined as *k* = 2, which was distinct and non-overlapping (Fig. 4A, B). Hence, two ubiquitination-associated molecular clusters of PAAD were established, which contained 115 cases in cluster 1 and 63 cases in cluster 2. The OS, PFI, DSS, and DFI were statistically different between two ubiquitination-related clusters (Fig. 4C–F). A lower ubiquitination pathway activity was displayed for patients with PAAD of cluster 2 than cluster 1 (Fig. 4G). The apparent differences of overall survival and pathway activity between two clusters indicated the vital role of ubiquitination in PAAD progression. Then, the discrepancies of clinicopathological traits were also investigated between two clusters. Tumor stage and histological grade also showed significant discrepancies between two clusters, suggesting the molecular clusters might be associated with the progression in PAAD (Fig. 4H). Then, we deeply explored the underlying mechanism of the different clinicopathological traits between two clusters. The volcano plot showed 2689 genes were upregulated, whereas 1656 genes were downregulated in cluster 2 compared to cluster 1 (|log2 fold change| > 1, FDR < 0.05) (Fig. 4I). Gene ontology enrichment analysis (GOEA) was performed with 2689 upregulated and 1656 downregulated genes using Cytoscape plug-in ClueGO, CluePedia, and yFiles Layout Algorithms. GOEA result is shown in Fig. 4J (*p* < 0.05), which discovered that the signaling pathways related to immune system process, epidermis development, and retinoic acid metabolism process showed significant differences. This result indicated TIME progression might be vital for ubiquitination-related poor prognosis.

### Cluster-based exploration of the discrepancy of TIME

**Fig. 5:**
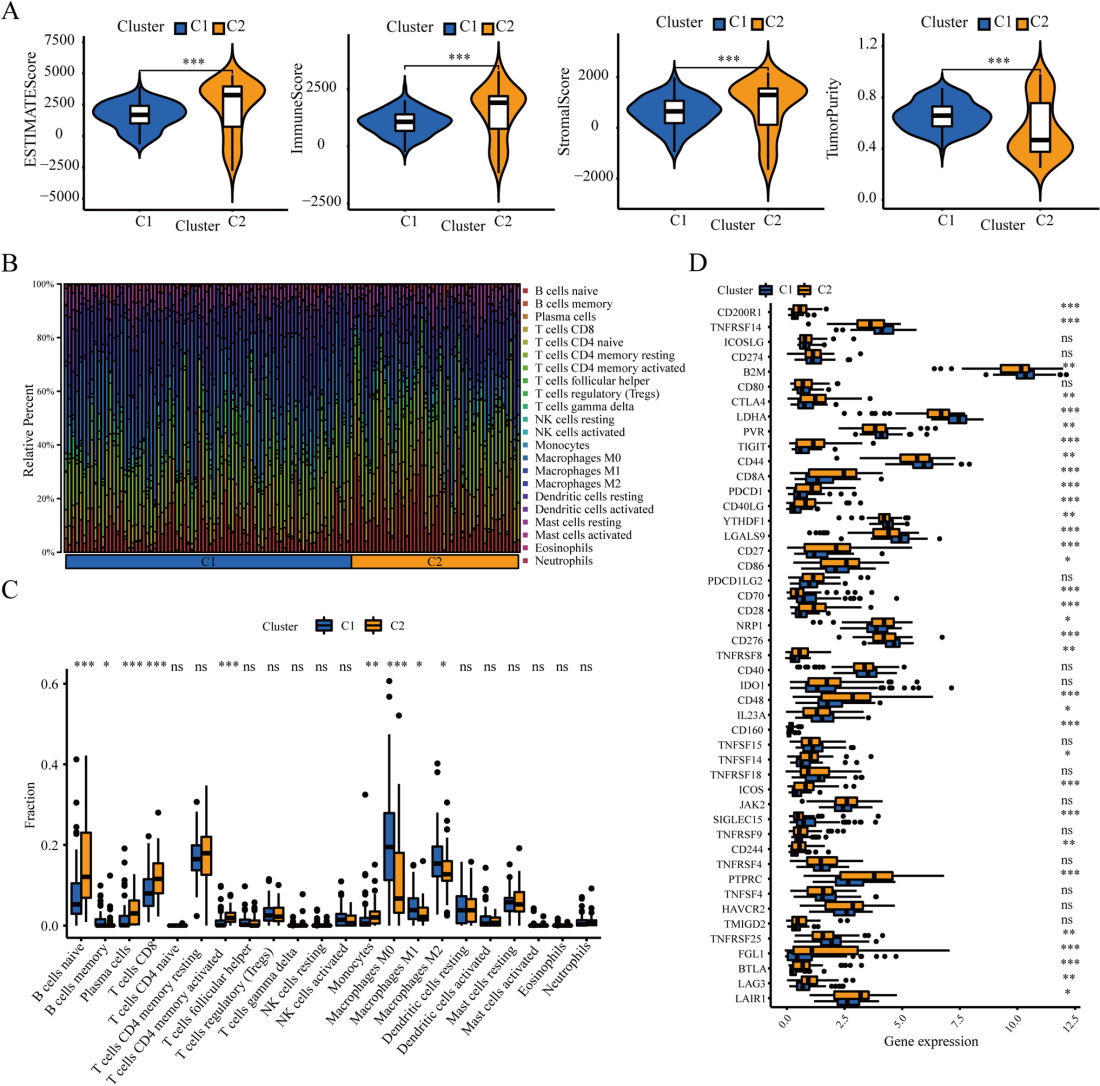
Exploration of the discrepancy of tumor immune microenvironment between two ubiquitination-related clusters. **A** EstimateScore, ImmuneScore, StromalScore, and tumor purity in two ubiquitination-related clusters. **B**, **C** The CIBERSOFT algorithm highlighted the traits of immunocyte distribution between cluster 1 and 2. **D** The differential expressions of 48 immune checkpoints genes in two ubiquitination-related clusters

To explore the discrepancy of TIME, “ESTIMATE” and “CIBERSORT” algorithms were performed with the help of R language. The results of “ESTIMATE” algorithm showed a higher ImmuneScore, StromalScore, and EstimateScore in cluster 2, whereas a stronger tumor purity in cluster 1 (Fig. 5A). The above results suggested that prognostic performance positively correlated to immune and stromal components in patients with PAAD. The “CIBERSORT” algorithm was utilized to display the proportional distribution of 22 subsets of tumor-infiltrated immune cells in patients of clusters 1 and 2, which revealed that the macrophage had an elevated infiltration in cluster 1, whereas the T cell had an enhanced infiltration in cluster 2 (Fig. 5B). This result was supported by the result of ICI by the “wilcox.test” function in R (Fig. 5C). Furthermore, the expression levels of 48 ICGs were depicted in two clusters (Fig. 5D). Taken together, these data hinted the key role of immune environment in prognostic discrepancy between ubiquitination-based molecular clusters.

### Construction and assessment of ubiquitination-related prognostic signature in the train cohort

**Fig. 6:**
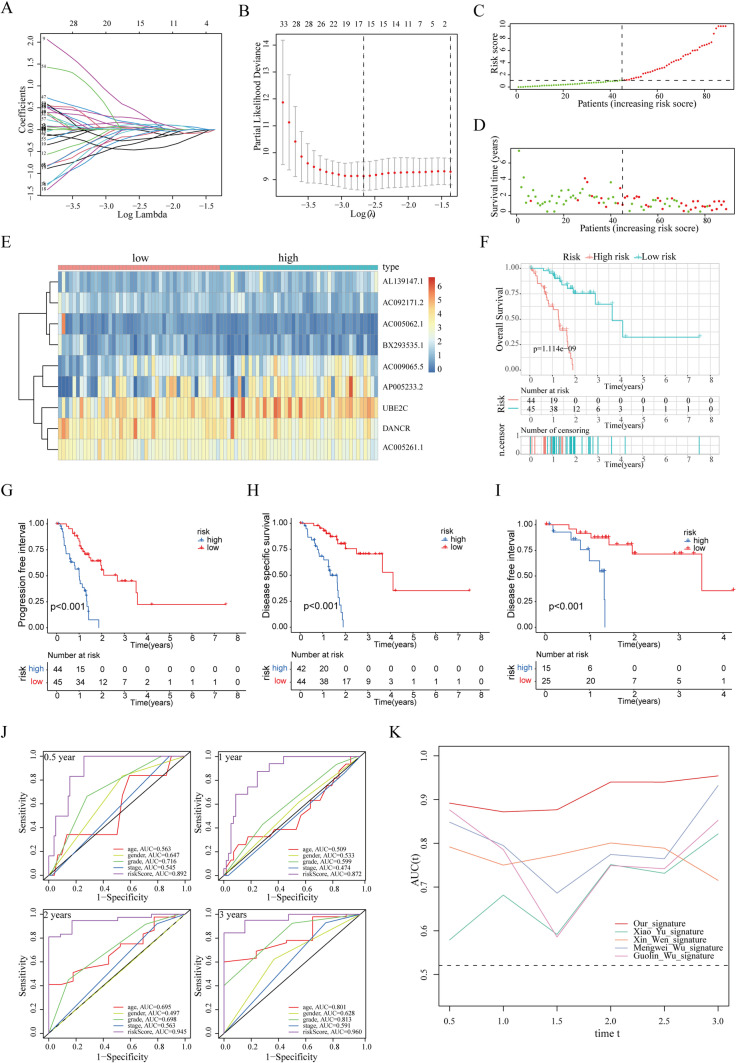
Construction and assessment of ubiquitination-related prognostic signature based on the train cohort. **A**, **B** The processing of LASSO algorithm. (**A** the trajectory changes of the independent variables; **B** the confidence interval of each lambda). **C** Sectionalization based on the median risk score. **D** Distribution characteristics of survival status in low-risk and high-risk populations. **E** Heatmap showed the molecular expression levels of 9 genes involved in the prognostic panel. **F–I** The discrepancies in clinical outcomes between low-risk and high-risk patients, including OS, PFI, DSS, and DFI. **J** Multi-index combined with ROC curve highlighted the superiority and great clinical application value of ubiquitination panel. **K** ROC curves of our URPS and other four prognostic signatures of PAAD

To accurately evaluate the survival probability in patients with PAAD, we constructed a novel URPS and validated it. In the train cohort, LASSO regression analysis of 79 candidate ubiquitination-related mRNAs and lncRNAs with traits of both differential expression and prognostic values was conducted to exclude collinearity and avert over-fitting of the prognostic model, which screened out 15 vital variables. Figure 6A presents the trajectory changes of the above independent variable parameters. The model construction using cross validation is presented in Fig. 6B. Multivariate Cox proportional hazards regression analysis of 15 vital variables was performed to establish the URPS that consisted of one mRNA and eight lncRNAs (Table 1).

**Table 1.** Multivariate Cox regression analysis to identify prognosis-related URGs

Gene	Coefficient	HR	HR.95L	HR.95H	*p* value
DANCR	−0.504471	0.6038251	0.3192458	1.1420817	0.1208055
AC005062.1	−1.447725	0.2351045	0.0533278	1.0364967	0.0557984
AL139147.1	1.0976127	2.9970027	1.3137959	6.836698	0.0090917
AC005261.1	−0.73841	0.4778731	0.2014814	1.1334184	0.0937875
AC009065.5	0.6279344	1.8737361	1.1244295	3.122372	0.015949
AC092171.2	−0.873169	0.4176259	0.1802551	0.967581	0.041666
UBE2C	0.558152	1.7474402	1.0741106	2.8428613	0.024583
AP005233.2	0.3331748	1.3953912	1.0281671	1.8937744	0.0324977
BX293535.1	−0.767486	0.4641787	0.2122659	1.0150566	0.0545393

Individual risk scores for PAAD in the train cohort were calculated using R’s “predict” function, and 89 PAAD patients were stratified into low- and high-risk categories according to a median risk score of 1.092 (Fig. 6C). The distributions of risk scores and clinical survival time were displayed for a subset of patients with PAAD (Fig. 6D), revealing a greater mortality in the high-risk grouping. The RNAs in our URPS were visualized as the heatmap that characterized the relative expressions of one mRNA and eight lncRNAs (Fig. 6E). Accordantly, survival analyses uncovered that patients with PAAD had a poor OS, PFI, DSS, and DFI in high-risk subpopulation with the help of the Kaplan-Meier method (Fig. 6F–I). This result suggested that our URPS could accurately distinguish the prognoses of patients with PAAD that distributed in different risk stratification.

Followingly, the efficiency and accuracy of our prognostic panel was further verified. The receiver operating characteristic (ROC) analysis showed the diagnostic efficacies of the clinical parameters and risk score. The area under the curve (AUC) values of risk score at 0.5 year, 1 year, 2 years, and 3 years were 0.892, 0.872, 0.945, and 0.960, respectively, which were higher than those of age, gender, grade, and stage (Fig. 6J). This result indicated that URPS-based risk score had a satisfied diagnostic performance better than age, gender, grade, and stage. In addition, the predictive performance of our URPS was contrasted to other four prognostic signatures of PAAD, which showed a better performance to predict survival of our URPS (Fig. 6K).

### The internal validation dataset verifies the robustness of the ubiquitination-related prognostic signature

To validate the robustness of our URPS, the risk scores of all 177 PAAD patients in the test1 cohort were computed depending on the same method as the train cohort. The median risk score in the train cohort was utilized to categorize patients with PAAD in the test1 cohort into low-risk and high-risk categories (Supplementary Figure 4A). Parallel to the train cohort, a higher risk score coupled with a higher death rate (Supplementary Figure 4B). The expression levels of these 9 genes in our URPS were coincident with those in the train cohort (Supplementary Figure 4C). Survival analysis of the Kaplan-Meier method exhibited a worse OS, PFI, DSS, and DFI in high-risk subgroup along with train cohort (Supplementary Figure 4D-G). Besides, time-dependent ROC analysis showed that the risk score had a better diagnostic performance (AUC = 0.727 for 0.5 year; AUC = 0.753 for 1 year; AUC = 0.779 for 2 years; AUC = 0.817 for 3 years) (Supplementary Figure 4H). Similarly, the survival possibility predicted by our URPS was also obviously stronger than other four prognostic signatures in the test1 cohort (Supplementary Figure 4I).

### The external validation dataset verifies the robustness of the ubiquitination-related prognostic signature

To further determine the validity and stability of our URPS, the model verification was conducted using 82 PAAD patients in the test2 cohort. The parameters and tools in the test2 cohort were coincident with those in the train cohort. Like the train and test1 cohorts, these high-scoring patients had more death events in the test2 cohort (Supplementary Figure 5A,B). And the expression trends of these 9 genes were accordant with the train and test1 cohorts (Supplementary Figure 5C). Survival analysis showed that coincident with the results in the train and test1 cohorts, there was a statistical difference of OS in the two subgroups (Supplementary Figure 5D). ROC analysis presented the AUC values of risk score, indicating the risk score in the test2 cohort also had a favorable diagnostic performance (AUC = 0.681 for 0.5 year; AUC = 0.764 for 1 year; AUC = 0.757 for 2 years; AUC = 0.883 for 3 years) (Supplementary Figure 5E).

### Independent prognostic performance of the ubiquitination-related prognostic signature

Risk scores and clinicopathological features in 177 PAAD samples were chosen to determine whether or not our URPS might behave as an independent prognostic indicator using variate Cox proportional hazards regression analysis. Independent prognostic indexes were identified as the risk score and cancer status in this study (risk score in univariate analysis: HR = 1.0578, 95% CI = 1.0329–1.0833, *p* < 0.001; risk score in multivariate analysis: HR = 1.0347, 95% CI = 1.0059–1.0642, *p* = 0.0176; cancer status in univariate analysis: HR = 4.6081, 95% CI = 2.1332–9.9540, *p* < 0.001; cancer status in multivariate analysis: HR = 3.0927, 95% CI = 1.3312–7.1853, *p* = 0.0087) (Table 2).

**Table 2:** Univariate and multivariate Cox regression analysis determined the independent prognostic performance of our risk score

	HR	HR.95L	HR.95H	*p* value
Univariate				
^a^stage	2.4455	1.4762	4.0512	0.0005
^b^grade	1.7026	1.1639	2.4906	0.0061
^c^tumor_diameter	1.2418	0.6909	2.2320	0.4692
^d^type_of_surgery	1.4723	1.0471	2.0702	0.0261
^e^residual_tumor	1.6925	0.9777	2.9298	0.0602
^f^radiation_therapy	0.3821	0.1620	0.9009	0.0279
^g^cancer_status	4.6081	2.1332	9.9540	0.0001
^h^history_of_pancreatitis	0.9945	0.3921	2.5224	0.9908
^i^Smoking_history	0.8730	0.7032	1.0839	0.2186
^j^riskScore	1.0578	1.0329	1.0833	<0.0001
Multivariate				
^a^stage	1.5099	0.7560	3.0160	0.2431
^b^grade	1.5199	0.9647	2.3945	0.0711
^c^tumor_diameter	1.3234	0.6936	2.5251	0.3952
^d^type_of_surgery	1.2330	0.8609	1.7659	0.2532
^e^residual_tumor	1.2787	0.6923	2.3618	0.4322
^f^radiation_therapy	0.4114	0.1684	1.0055	0.0514
^g^cancer_status	3.0927	1.3312	7.1853	0.0087
^h^history_of_pancreatitis	0.9527	0.3557	2.5512	0.9231
^i^Smoking_history	0.8262	0.6477	1.0538	0.1241
^j^riskScore	1.0347	1.0059	1.0642	0.0176

^*a*^*stage*, stage I, stage II, stage III, stage IV; ^*b*^*histologic_grade*, G1, G2, G3, G4; ^*c*^*maximal tumor diameter*, <3.5, ≥3.5; ^*d*^*type_of_surgery*, distal pancreatectomy, total pancreatectomy, Whipple, other method; ^*e*^*residual_tumor*, R0, R1, R2; ^*f*^*radiation_therapy*, no, yes; ^*g*^*cancer_status*, tumor free, with tumor; ^*h*^*history_of_pancreatitis*, no, yes; ^*i*^*Smoking_history*, I/II/III/IV/V; ^*j*^*riskScore*, risk scores of each patient were calculated with the help of “predict” function in R

### URPS-based pathway annotation

**Fig. 7:**
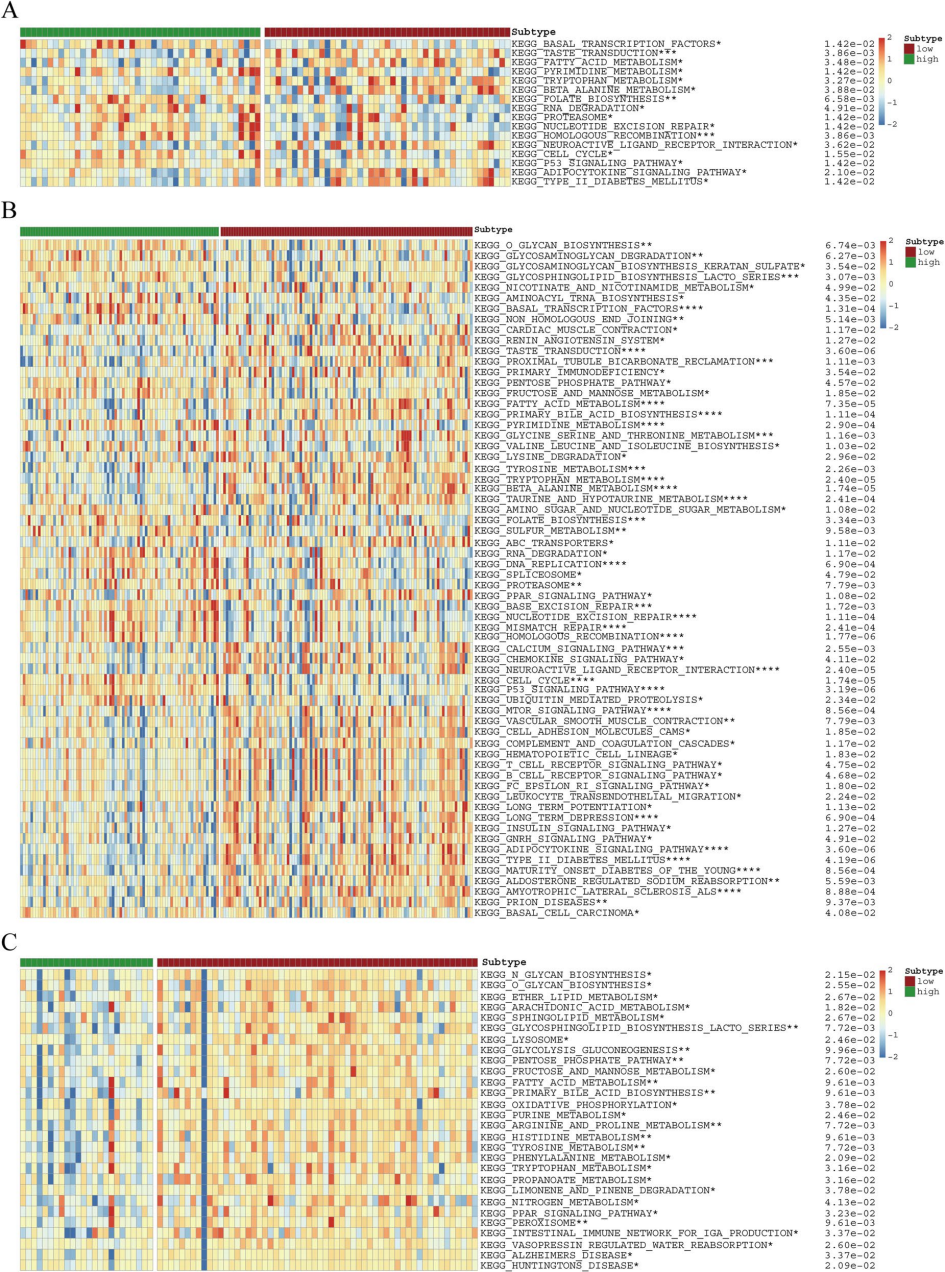
Inherent molecular heterogeneity in high- and low-risk patients. **A–C** GSEA showed the discrepancies in the activities of KEGG-derived pathways between different risk subgroups in the training, test1, and test2 cohorts

**Fig. 8:**
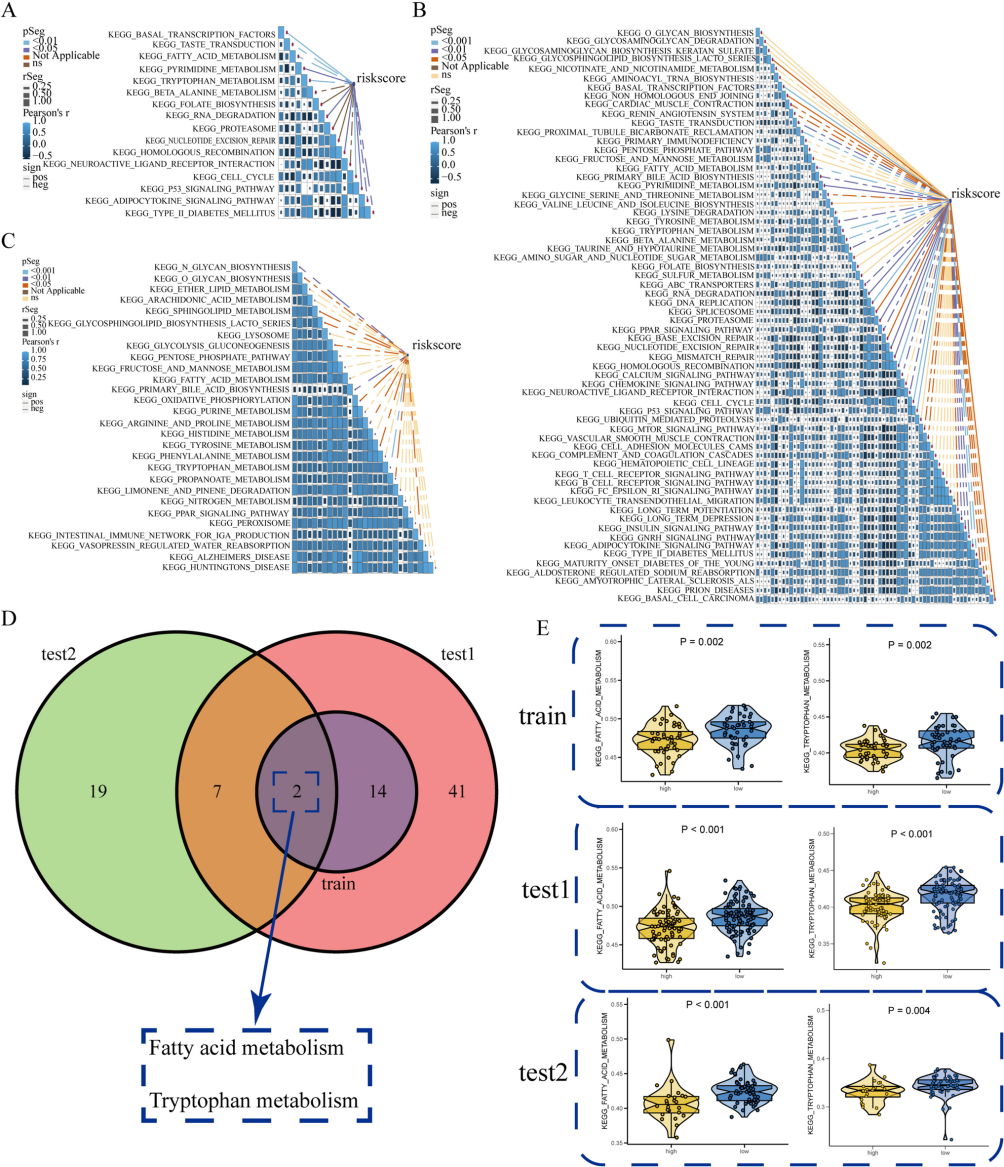
The correlation between differential tumor-related pathways and risk score. **A–C** Pearson correlation analysis showed the correlation between risk score and the enrichment score of tumor-related pathways in the training and test cohorts. **D** Two pathways screened by the intersection of tumor-related pathways among the training and test cohorts. **E** The discrepancy of fatty acid and tryptophan metabolism scores computed by ssGSEA in the training and test cohorts (ordinate: metabolism-related pathway scores calculated by ssGSEA. A higher metabolism score indicates a higher metabolism activity)

To explore the discrepancy of potential signaling pathways between low-risk and high-risk crowds, we performed ssGSEA in the train and test cohorts. The differential abundance of pathway activities between low-risk and high-risk crowds in the training and test cohorts is depicted in Fig. 7A–C. The correlation between these pathway activities and risk scores is shown in Fig. 8A–C. Interestingly, numerous metabolism-related pathways exhibited significant difference between low-risk and high-risk crowds. Of note, the pathway activities of both fatty acid and tryptophan metabolism were obviously downregulated in high-risk crowd compared to low-risk crowd in the train and test cohorts (Fig. 8D, E). These results suggested the close association between ubiquitination modification and metabolic reprogramming, especially of fatty acid and tryptophan metabolism.

### URPS-based characterization of the TIME

**Fig. 9:**
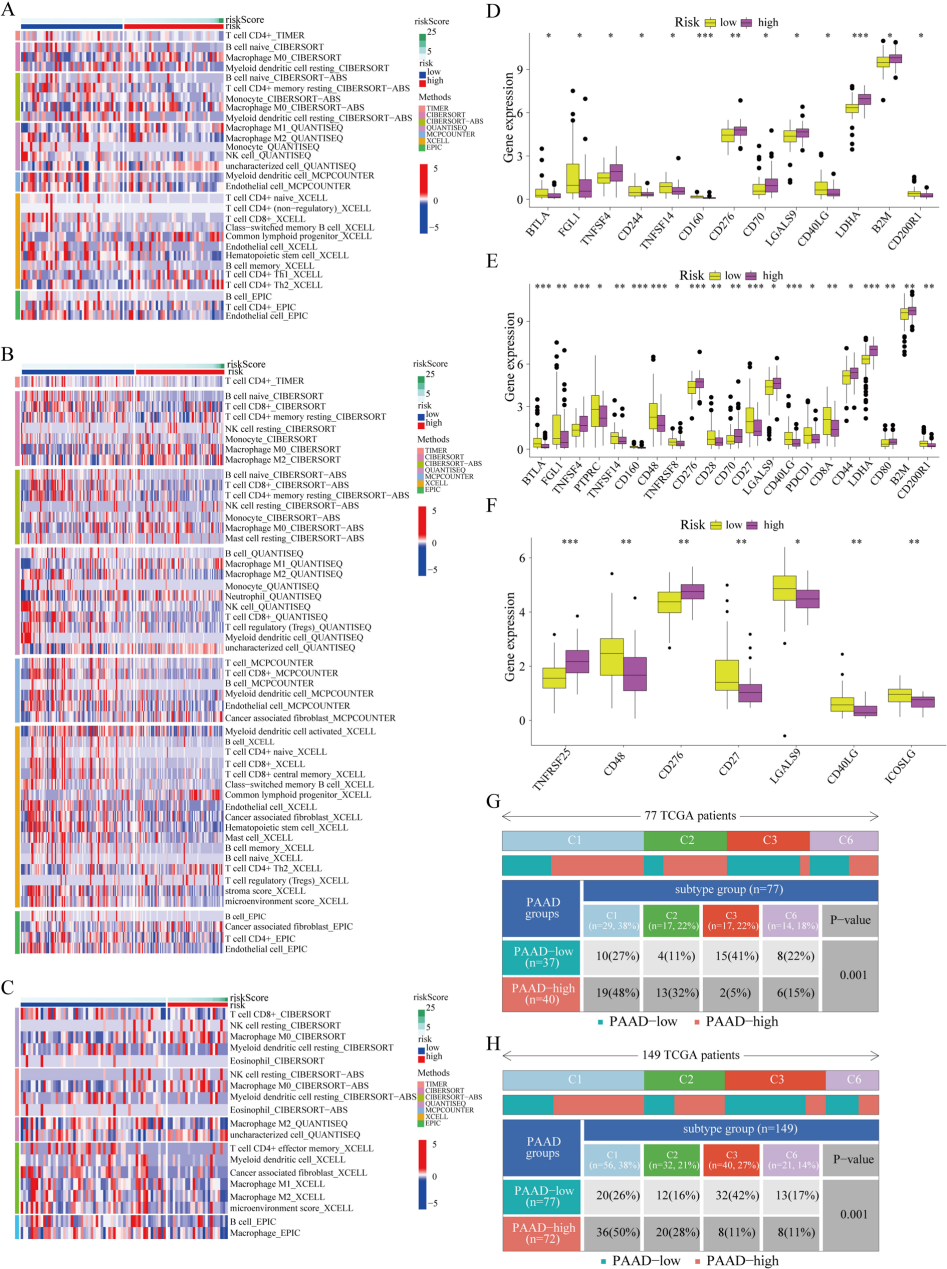
The differences of immune characteristics between low-risk and high-risk subgroups. **A–C** The heatmaps showed immune cell infiltrations in different risk subgroups based on the training and test cohorts. **D–F** The gene expressions of immune checkpoints in low-risk and high-risk subgroups based on the training and test cohorts. **G**, **H** The distributions of immune subtypes in low-risk and high-risk subgroups based on the training and test1 cohorts

Using TIMER, CIBERSORT, CIBERSORT-ABS, QUANTISEQ, MCPCOUNTER, XCELL, and EPIC algorithms, the difference in immunocyte infiltration between low-risk and high-risk populations was analyzed. The heatmap about ICI showed the fewer infiltrations of CD4^+^ T cells, CD8^+^ T cells, natural killing (NK) cells, and naive B cells in high-risk subgroup from the train cohort, which was verified by the results of ICI in the test cohorts (Fig. 9A–C). Given the significance of immune checkpoint inhibitor-based immunotherapies for cancer, the gene expression of common ICGs between two subgroups is shown in Fig. 9D–F. Interestingly, only CD276 and CD40LG exhibited similar expression trend in all the three cohorts, indicating CD276 and CD40LG might function as potential therapeutic targets of low-risk and high-risk populations. Subsequently, we investigated the difference of immune subtypes between two subgroups. For both the train and test1 cohorts, wound healing (C1) and IFN-γ dominant (C2) were the main immune subtypes in high-risk subgroup, while inflammatory (C3) and TGF-β dominant (C6) were the primary immune subtypes in low-risk subgroup (Fig. 9G, H).

### URPS-based characterization of the tumor mutation burden

**Fig. 10:**
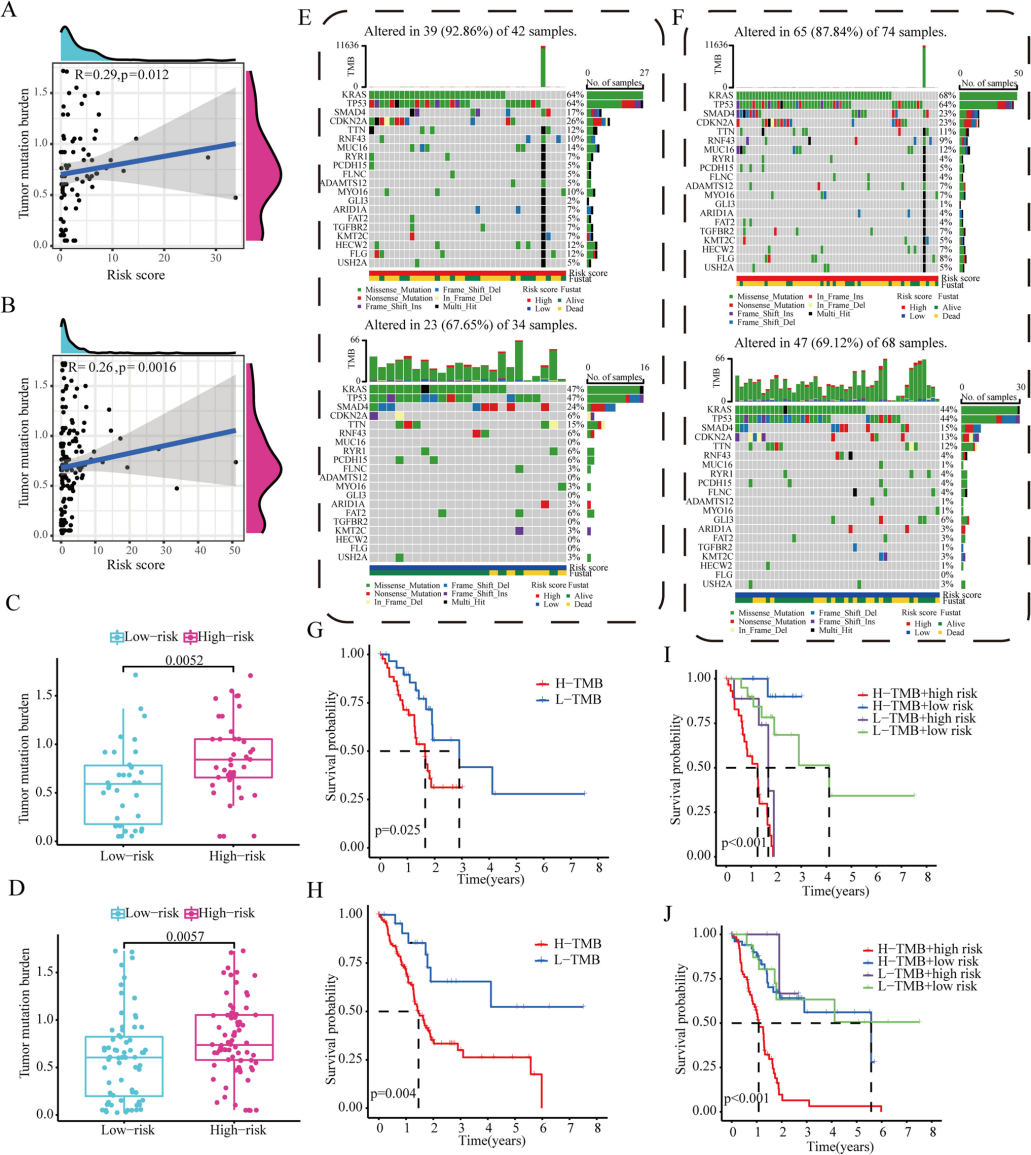
The association between ubiquitination-related risk score and TMB. **A**, **B** Spearman correlation analysis showed the close association of ubiquitination-related risk score and TMB in the training and test1 cohorts. **C**, **D** The discrepancies in the levels of TMB in different risk subgroups based on the training and test1 cohorts. **E**, **F** Top 20 mutation gene traits were displayed in the heatmap in the training and test1 cohorts. **G**, **H** Correlation between TMB levels and clinical outcomes of PAAD patients in the training and test1 cohorts. **I**, **J** Survival analysis of OS for patients stratified by the TMB and risk scores

Considering the prognostic and clinical values of TMB, the correlation between risk score and TMB score was researched based on the mutation information of PAAD patients from the train and test1 cohorts. Spearman correlation analysis revealed that risk scores positively correlated to TMB scores in the train and test1 cohorts (train cohort: *p* = 0.012; test1 cohort: *p* = 0.0016) (Fig. 10A, B). Similarly, the TMB scores of high-risk segment were visibly greater than those of the low-risk subpopulation (Fig. 10C, D). There was a visibly higher mutation quantity of genes in high-risk subgroup, which centrally covered KRAS, tumor protein p53 (TP53), and SMAD4 (Fig. 10E, F). The patients with PAAD that acquired high TMB scores had a shorter survival period than individuals with low TMB values (Fig. 10G, H). To further analyze the synergistic or antagonistic relationship between TMB score and risk score in survival prediction, patients with PAAD were stratified on the basis of these two scores for subsequent survival analysis, which revealed a worse survival time in patients that got high TMB and risk scores than the others (Fig. 10I, J).

### Targeted drug sensitivity prediction

**Fig. 11:**
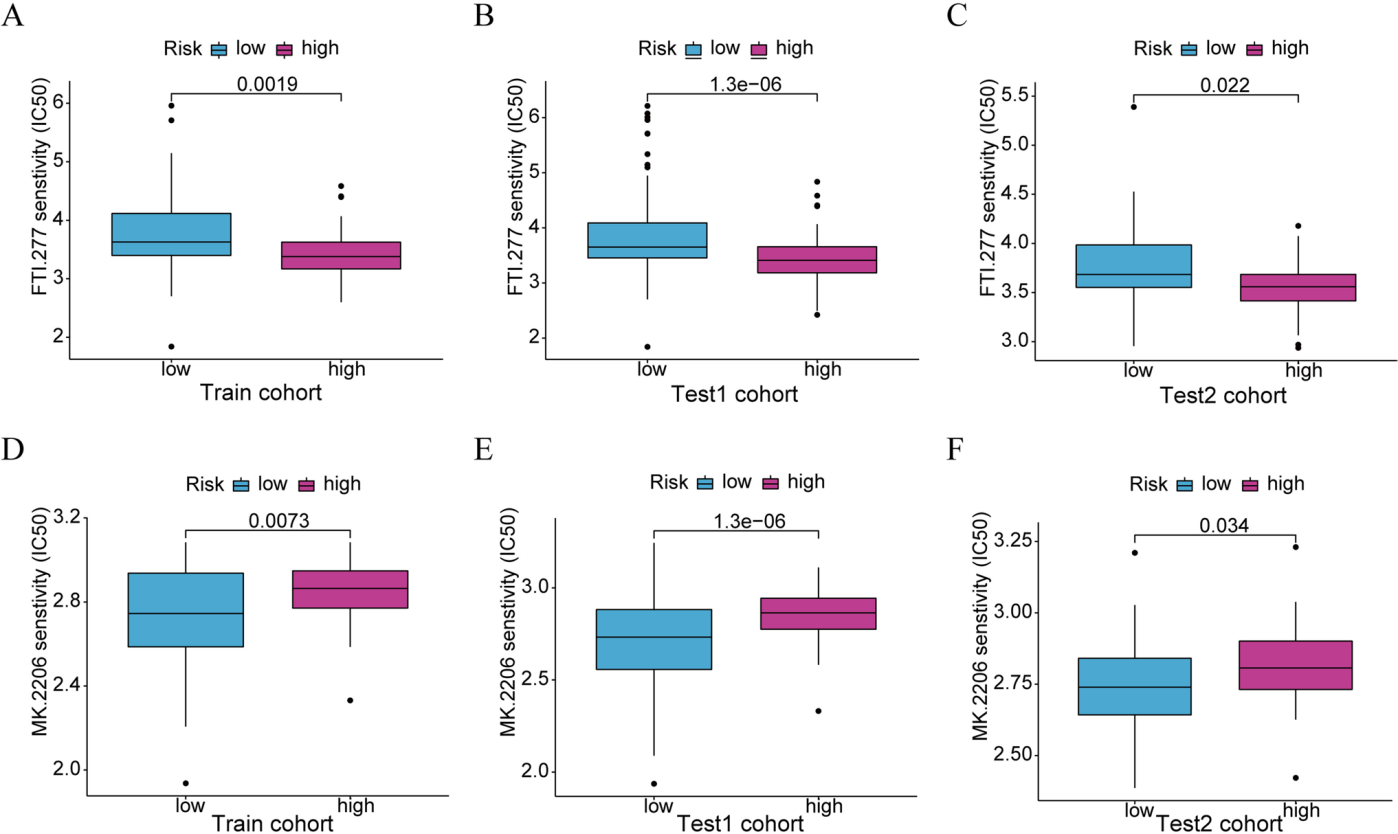
Targeted drug sensitivity prediction between low-risk and high-risk subgroups. **A–C** The box plots of the estimated IC50 for FTI-277 in the training and test cohorts, respectively. **D–F** The box plots of the estimated IC50 for MK-2206 in the training and test cohorts, respectively

Taking into account the clinical values of the molecularly targeted therapy in PAAD, we screened the potentially sensitive drugs for low-risk and high-risk patients depending on the drug sensitivity evaluated using the “pRRophetic” package in the train and test cohorts. Our findings revealed that FTI-277 had a better curative effect in high-risk patients, while MK-2206 had a superior therapeutic effect in low-risk patients (Fig. 11A–F).

### Verification of the expression levels of nine URGs in PAAD cell lines and clinical samples

**Fig. 12:**
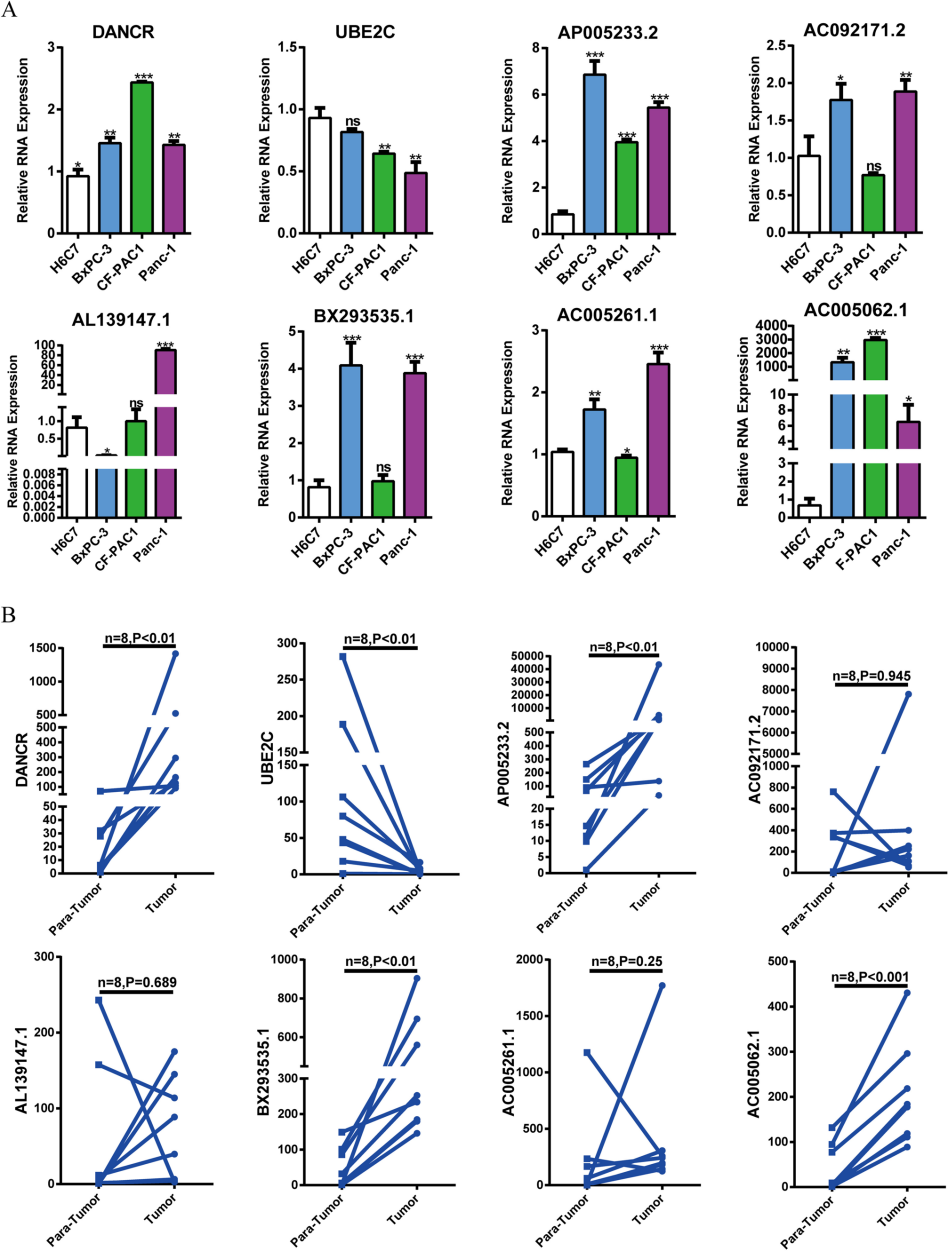
Verification of the expression levels of eight URGs in PAAD cell lines and clinical samples. **A** The qRT-PCR assays showed the expression levels of DANCR, UBE2C, AC005062.1, AL139147.1, AC005261.1, AC092171.2, AP005233.2, and BX293535.1 in three PAAD cell lines (BxPC-3, CF-PAC1, Panc-1) and the normal pancreas cell line H6C7. **B** The qRT-PCR assays showed the expression levels of DANCR, UBE2C, AP005233.2, AC092171.2, AL139147.1, BX293535.1, AC005261.1, and AC005062.1 in PAAD and para-tumor samples

To clarify the expression traits of nine URGs in PAAD, qRT-PCR was used to measure the RNA expression levels of one mRNA and eight lncRNAs involved in the signature (i.e., mRNA: UBE2C; lncRNA: DANCR, AP005233.2, AC092171.2, AL139147.1, BX293535.1, AC005261.1, AC005062.1, and AC009065.5). These nine RNAs were measured in three PAAD cell lines (BxPC-3, CF-PAC1, Panc-1) and the normal pancreas cell line H6C7. We also collected 8 paired PAAD samples and para-tumor samples to examine the differential expression of above nine URGs. Among above RNAs, eight RNAs could be detected in both cell and tissue levels (Fig. 12A, B). As for DANCR, UBE2C, AP005233.2, BX293535.1, and AC005062.1, qRT-PCR assays of both cell lines and clinical samples displayed the same overall trends (Fig. 12A, B). Of note, qRT-PCR assays showed that the RNA expressions of AC005062.1 were largely enhanced in both cell lines and tissues, indicating its irreplaceable value in ubiquitination-related prognosis of patients with PAAD (Fig. 12A, B).

## Discussion

PAAD, a highly fatal malignancy, is characterized by early metastasis and resistance to anti-cancer therapy, which has been the seventh leading death from cancer worldwide (Ryan et al. 2014; Bray et al. 2018). Despite the rapid development of diagnosis and therapeutic strategies for malignant tumors, PAAD patients are often diagnosed at a late stage and benefit little from current treatments. Therefore, the development of biomarkers for early diagnosis and risk assessment of pancreatic cancer has important clinical significance (Lu et al. 2023; Zheng et al. 2023). Recently, the role of ubiquitination-related regulators in the occurrence and outcome of pancreatic cancer has been deeply researched, which can influence the occurrence, progression, metastasis, and treatment response of various cancers (Ma et al. 2020; Sun et al. 2020; Chen et al. 2021). Ubiquitination-related prognostic signatures have been developed for estimating the prognosis of several types of cancers (Cai et al. 2021; Wu et al. 2021; Zhou et al. 2021). In our research, the expression characteristics and prognostic performances of URGs in PAAD were systematically explored to classify different ubiquitination-related clusters and a novel prognostic signature was precisely established for disease stratification. To our knowledge, this is the first report to identify an ubiquitination-based mRNA-lncRNA prognostic panel in PAAD using bioinformatics.

Pan-cancer analysis uncovered the significant role of URGs across various cancers, which was profoundly explored based on the multi-omics data. It is the first time to systemically display the gene expression, gene variation, OS, and signaling pathways of URGs across various cancer types, indicating the impressive regulation of ubiquitination-related immune response on cancers. In this study, we conducted more in-depth research for PAAD. The location of normal and malignant cells in PAAD tissues was identified in the first portion of the research by the scRNA-seq analysis. Significant differences of the activation of ubiquitination pathways and the expression traits of URGs were detected between normal and malignant cells, also suggesting the close association of ubiquitination and PAAD progression. Next, the ubiquitination-related clusters of PAAD were explored using consensus clustering analysis and risk analysis. In cluster analysis, PAAD patients were successfully divided into two clusters with distinct clinical outcomes, ubiquitination scores, and immunocyte infiltration status. Patients with PAAD of cluster 2 were accompanied by favorable clinical outcomes and lower ubiquitination pathway activities, further validating the risky roles of ubiquitination in PAAD. More importantly, there was a remarkable correlation between ubiquitination-based clusters and clinicopathological characteristics (i.e., tumor stage and tumor grade), indicating that ubiquitination closely correlated to the malignant degree of PAAD. Meanwhile, GOEA results suggested that the different prognoses between two ubiquitination-related clusters were greatly impacted by the abundance of ICI, particularly macrophage and T cell. Thus, URGs might occupy a dominating status in the occurrence and progression of patients with PAAD through regulating the local immune microenvironment.

The risk analysis showed that a novel URPS was established based on differentially expressed candidate genes with prognostic values from the train cohort using LASSO-Cox regression analysis, including AL193147.1, AC092171.2, AC005062.1, BX293535.1, AC009065.5, AP005233.2, UBE2C, DANCR, and AC005261.1. According to the current literature search, there are three genes having been investigated, but the other six are not reported till now. UBE2C is a necessary component of the ubiquitin proteasome system, involved in the degradation of anaphase-promoting complex (APC/C) target proteins (Jin et al. 2008; Meyer and Rape 2011). The biological functions of UBE2C include ubiquitin conjugation, degradation of major proteins regulating cell cycle progression, and regulation of mitotic spindle checkpoint (Xie et al. 2014). The mRNA and/or protein levels of UBE2C are aberrantly enhanced in pancreatic cancer with dismal clinical outcomes (Shi et al. 2020; Zhu et al. 2021). However, our confirmatory experiments showed the mRNA expression of UBE2C between para-tumor and tumor tissues in patients with PAAD was opposite to the results in the above literature, indicating the gene-expressing heterogeneity in pancreatic cancer. Additionally, DANCR plays a key role in pancreatic cancer via modulating tumor cell proliferation and immune response (Hu et al. 2020; Tang et al. 2020). Studies show that AP005233.2, a highly expressed lncRNA in tumor tissue, associates with the prognosis of lung adenocarcinoma and intrahepatic cholangiocarcinoma (Qi et al. 2019; Zou et al. 2021). The expressional tendencies of DANCR and AP005233.2 in our experiments were consistent with these literatures. Of note, the RNA expression of AC005062.1 in the entire three PAAD cell lines was elevated several thousandfold, suggesting its extremely valuable for prognostic prediction and targeted therapy of pancreatic cancer in subsequent research. Patients with PAAD had statistically significant differences of survival time between low-risk and high-risk populations, indicating the excellent prognostic discrimination of our panel and the vital part of these URGs in pancreatic cancer prognosis.

Subsequently, we explored the underlying mechanisms of this different prognosis of patients with PAAD between low-risk and high-risk populations. URPS-based pathway annotation displayed fatty acid and tryptophan metabolisms were detected to function as protective roles in the ubiquitination progression of patients with PAAD. The fatty acid metabolism is obviously altered in cancer cell that accelerates tumor progression (Koundouros and Poulogiannis 2020). De novo fatty acid synthesis is essential for tumor proliferation, membrane generation, and tumor-promoting signaling molecules (Currie et al. 2013; Koundouros and Poulogiannis 2020). It has been reported that fatty acid metabolism makes a deep contribution to pancreatic cancer malignancy (Downes et al. 2020). Inversely, omega-3 polyunsaturated fatty acids (PUFAs) inhibit tumor deterioration via reducing the local inflammation, inducing cancer cell apoptosis, and suppressing tumor angiogenesis (Torres et al. 2018). In our study, the decreased fatty acid metabolism in high-risk subgroup based on ubiquitination-related genes indicated the decrease of omega-3 PUFAs metabolism might be responsible for ubiquitination-related poor outcome in patients with PAAD. In the tumor microenvironment, tryptophan metabolism-related enzymes produced by tumor cells and tryptophan metabolites are involved in the induction of immune tolerance (Mellor and Munn 2004). In addition, tryptophan metabolism induced by IDO1 enzyme in pancreatic cancer cell is the source of one-carbon units for pancreatic stellate cells, which accelerates the development of PAAD (Newman et al. 2021). By contrast, our analysis showed that the tryptophan metabolism was reduced in high-risk subgroup. The potential reason for this opposite result was that some signaling molecules among tryptophan metabolism were involved in the inhibition of tumor progression that were yet discovered or only tumor heterogeneity. The above results suggested that the tumor cell proliferation and immune response regulated by fatty acid and tryptophan metabolism might participate in ubiquitination-related poor prognosis of pancreatic cancer patients.

Due to the infiltration of immunosuppressive leukocytes and minimal infiltration of antitumor T cells, pancreatic cancer is usually deemed to be immunosuppressed (Vonderheide and Bayne 2013). It is worth noting that ICI and immune checkpoints had statistical differences between low-risk and high-risk subgroups. Patients with PAAD in high-risk subgroup had fewer infiltrations of CD4^+^ T cells, CD8^+^ T cells, NK cells, and naive B cells. By retrieving literatures, these immune cells can detect and eradicate tumor cells via different mechanisms (Borst et al. 2018; Terrén et al. 2019; Philip and Schietinger 2021). Research has revealed that survival time was significantly longer in PAAD patients with high levels of CD4^+^ and/or CD8^+^ T cells (Carstens et al. 2017). The lack of CD4^+^ T cells, CD8^+^ T cells, and NK cells that results in decreased tumor cell clearance is responsible for ubiquitination-related unsatisfactory prognosis in pancreatic cancer patients. Besides, the gene expressions of CD276 and CD40LG were statistically different between these two subgroups. CD276, an immune checkpoint molecule, regulates cell proliferation, invasion, and migration of malignant tumors (Liu et al. 2021). The transmembrane protein CD40LG, as a member of the tumor necrosis factor (TNF) gene superfamily, is a ligand of CD40 and largely generated by activated T cells (Laman et al. 2017). In breast cancer, CD40LG has been developed as a key prognostic gene associated with the tumor microenvironment (Yuan et al. 2021). More than that, PAAD patients in different risk stratification had a significant difference of immune subtypes. Thus, it can be seen that TIME is vital for the malignant degree and ubiquitination-related prognosis of PAAD.

Gene mutation exerts a pivotal effect on the efficacy of targeted drug therapy for pancreatic cancer (Liu and Qian 2022; Yuan et al. 2022). We also investigated the discrepancies of gene mutation and targeted drug between these two subgroups. The waterfall plot uncovered a visibly higher mutation quantity of genes in high-risk population, primarily including KRAS, p53, and SMAD4. KRAS mutation contributes to tumor inception, and the mutations of p53, SMAD4, and CDKN2A are rate-limiting events for tumor progression and metastasis (Hustinx et al. 2005; Qian et al. 2020). Interestingly, the targeted drug sensitivity analysis displayed that high-risk patients were more able to benefit from FTI-277. FTI-277, as one of farnesyltransferase (FTase) inhibitors, can regulate Ras signaling pathway via inhibiting Ras membrane association (Cox et al. 2015). The Ras family members of 21-kDa GTPases serve as molecular switches of signaling pathways of cell survival, proliferation, and immune response (Carbone et al. 2005; Hancock and Parton 2005). The ubiquitination-associated Ras signaling pathway, especially KRAS mutation, strongly might participate in the regulation of FTI-277-targeted therapy for pancreatic cancer. The molecular mechanism that how Ras signaling pathway influences the targeted therapy of FTI-277 by interacting with ubiquitination regulators is still further explored.

Our research has a few limitations that should be acknowledged. Firstly, this study belonged to retrospective research and was performed mainly on the basis of public databases. The number of PAAD patients from TCGA, ICGC, and GTEx databases was relatively small for establishing the ubiquitination-related prognostic model. Thus, a prospective clinical study with large samples is required for the validation of the predictive performance of our prognostic model. Secondly, further investigation of molecular mechanism is required to examine the role of the nine URGs in the occurrence and progression of PAAD.

## Conclusion

In this study, we found an essential association between ubiquitination and pancreatic cancer through pan-cancer and scRNA-seq analyses. The clusters of PAAD patients based on URGs demonstrated that ubiquitination cloud determine the poor prognosis of pancreatic cancer. Furthermore, we constructed a novel ubiquitination-related mRNA-lncRNA prognostic panel with outstanding prediction capacity. Depending on this panel, we deeply investigated the underlying mechanisms of different prognosis between two risk-ranking populations in pancreatic cancer patients, uncovering the metabolic reprogramming, TIME, TMB, and targeted drug therapy strongly associated with ubiquitination-related poor prognosis. This provides a new thinking for creating the effective strategies of diagnostic program, prognostic evaluation, and targeted drug therapy in pancreatic cancer.

## Supplementary information

ESM 1 (XLSX 30 kb)

ESM 2 (XLSX 29 kb)

ESM 3 (XLSX 9 kb)

ESM 4 (DOCX 12 kb)

ESM 5 (XLSX 44 kb)

ESM 6 Supplementary Figure 1 Single cell RNA sequencing analysis of 24 PAAD samples. (A) Data quality control and cleaning. (B) The link between UMI and mRNA abundance, as well as the association between UMI/mRNA abundance and mitochondrial gene. (C) Data from scRNA-seq analyses identified the top 50 PCs in a principal component analysis. (PNG 1420 kb)

High resulotion image (TIF 7133 kb)

ESM 7 Supplementary Figure 2 The enrichment scores of cancer-associated pathways and URGs in the single cell levels of pancreatic cancer. (A) The distributions of cancer-associated pathways in malignant and non-malignant cells of 24 PAAD samples. (B) The expression levels of different URGs in malignant and non-malignant cells of 24 PAAD samples. (PNG 1164 kb)

High resulotion image (TIF 17582 kb)

ESM 8 Supplementary Figure 3 Construction of mRNA-lncRNA co-expressed network and scanning of specific ubiquitination-related mRNA and lncRNA with prognostic values. (A) Construction of mRNA-lncRNA co-expressed network. Pearson correlation analysis showed the correlation between 64 ubiquitination-related mRNAs and 10119 lncRNAs (|R| >0.4 and P<0.01). (B) Identification of differentially expressed mRNAs and lncRNAs with prognostic values. (C) The heatmap displayed the expression distributions of specific mRNAs and lncRNAs in PAAD and normal samples. (D) The forest plot displayed the prognostic performances of specific mRNAs and lncRNAs in PAAD. (PNG 2064 kb)

High resulotion image (TIF 10574 kb)

ESM 9 Supplementary Figure 4 The internal dataset of test1 cohort verifies the effectiveness of the ubiquitination-related prognostic panel. (A) Sectionalization of different risk subpopulations in the test1 cohort. (B) Correlation between risk score and clinical outcomes in the test1 cohort. (C) The distributions of panel genes in different risk subpopulations in the test1 cohort. (D-G) Survival analysis of OS, PFI, DSS, and DFI in different risk subpopulations. (H) ROC curves showed the AUC values of clinical features and risk scores in the test1 cohort. (I) ROC curves of our URPS and other four prognostic signatures of PAAD. (PNG 991 kb)

High resulotion image (TIF 7071 kb)

ESM 10 Supplementary Figure 5 The external dataset of test2 cohort verifies the robustness of the ubiquitination-related prognostic signature. (A) Sectionalization of different risk subpopulations in the test2 cohort. (B) Correlation between risk score and clinical outcomes in the test2 cohort. (C) The distributions of panel genes in different risk subpopulations in the test2 cohort. (D) Survival analysis of OS in different risk subpopulations. (E) ROC curves showed the AUC values of clinical features and risk score in the test2 cohort. (PNG 352 kb)

High resulotion image (TIF 4208 kb)

## Data Availability

The datasets analyzed during the present investigation as well as the original data used in this study are accessible upon reasonable request from the corresponding author.
