# Supplementary material for: A prospective prognostic signature for pancreatic adenocarcinoma based on ubiquitination-related mRNA-lncRNA with experimental validation in vitro and vivo
Source: Funct Integr Genomics. 2023 Aug 4;23(3):263. doi: 10.1007/s10142-023-01158-1 (PMC10403435; doi:10.1007/s10142-023-01158-1)
Supplement: Supplementary file 4 — (DOCX 12 kb) [file 10142_2023_1158_MOESM4_ESM.docx]

The datasets, codes, and qRT-PCR original data used in this study can be acquired by clicking the link below: <https://pan.baidu.com/s/1spg2Xxq37AHTP6uHfejeHA?pwd=3n6r>
