# Supplementary figures and images for: A prospective prognostic signature for pancreatic adenocarcinoma based on ubiquitination-related mRNA-lncRNA with experimental validation in vitro and vivo
Source: Funct Integr Genomics. 2023 Aug 4;23(3):263. doi: 10.1007/s10142-023-01158-1 (PMC10403435; doi:10.1007/s10142-023-01158-1)

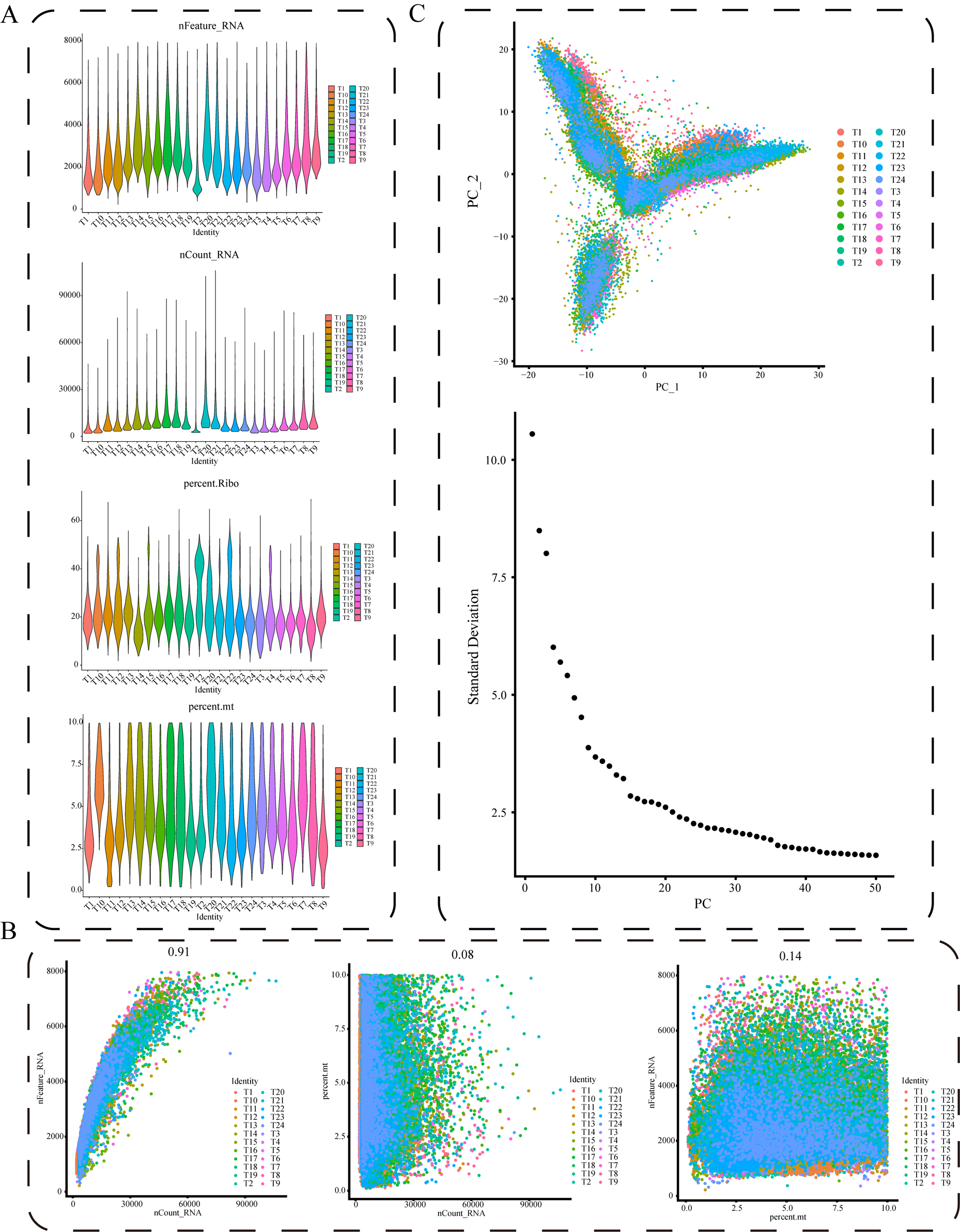

Supplement: Supplementary file 6 — Supplementary Figure 1 Single cell RNA sequencing analysis of 24 PAAD samples. (A) Data quality control and cleaning. (B) The link between UMI and mRNA abundance, as well as the association between UMI/mRNA abundance and mitochondrial gene. (C) Data from scRNA-seq analyses identified the top 50 PCs in a principal component analysis. (PNG 1420 kb) [file 10142_2023_1158_Fig13_ESM.png]

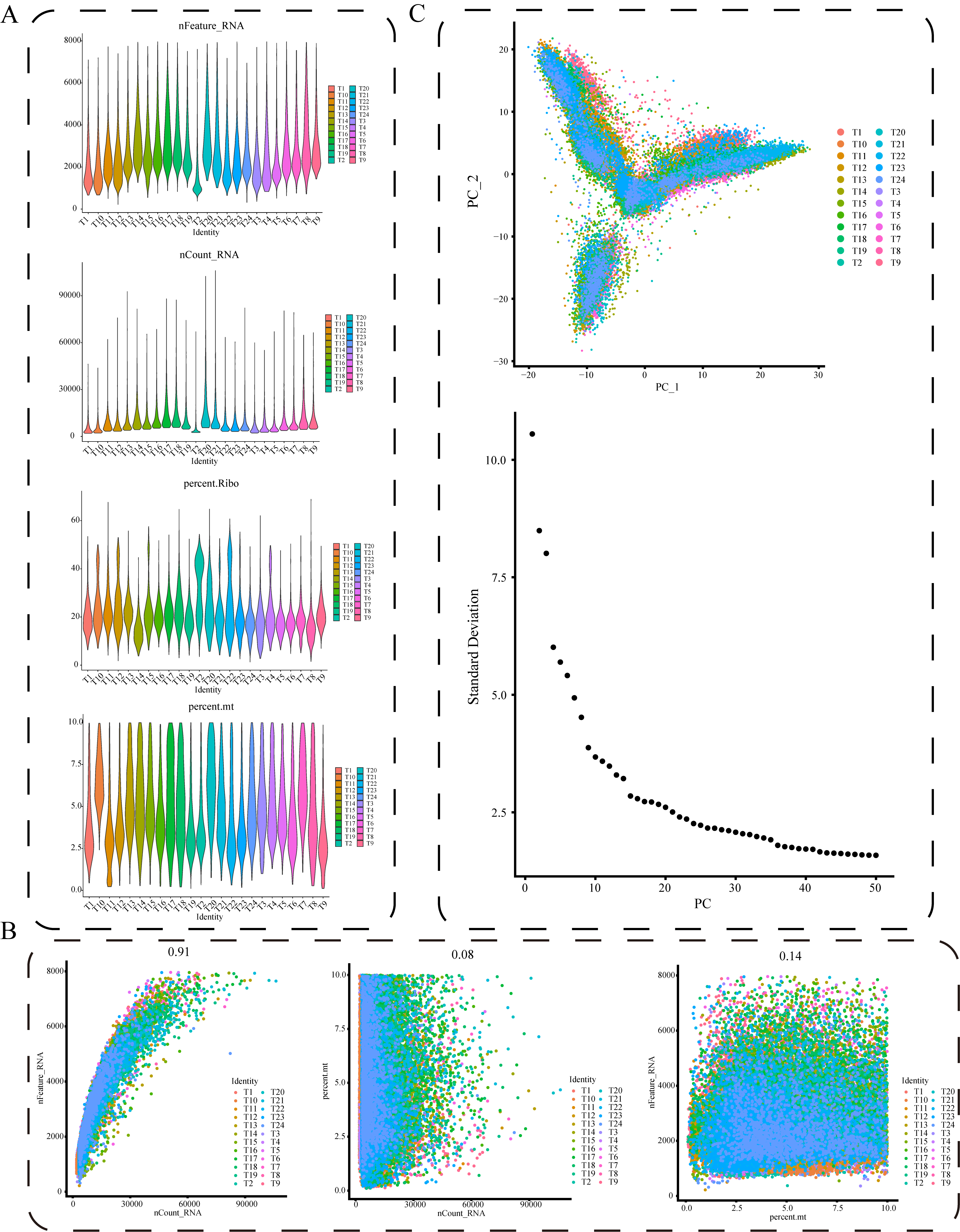

Supplement: Supplementary file 7 — High resulotion image (TIF 7133 kb) [file 10142_2023_1158_MOESM6_ESM.tif]

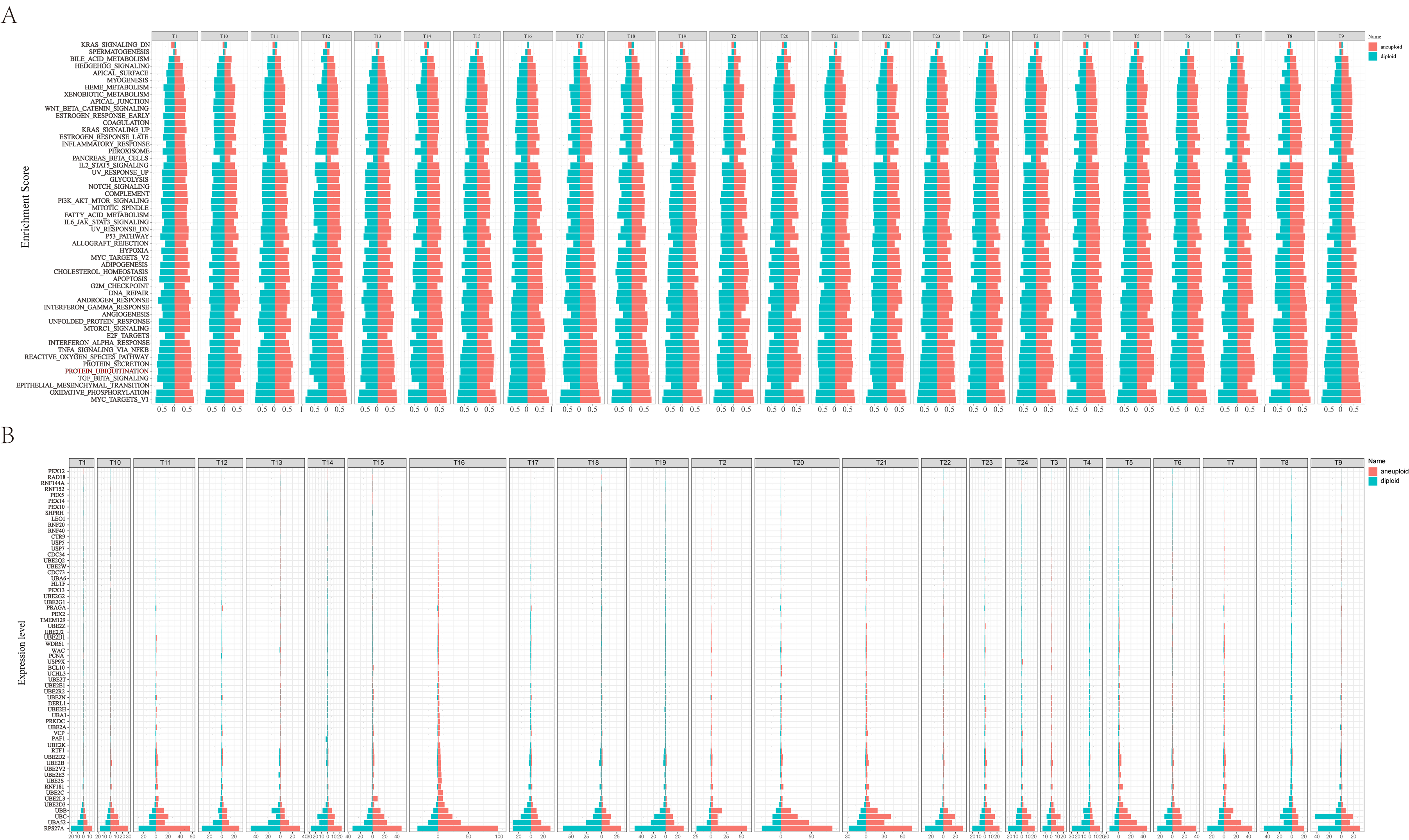

Supplement: Supplementary file 8 — Supplementary Figure 2 The enrichment scores of cancer-associated pathways and URGs in the single cell levels of pancreatic cancer. (A) The distributions of cancer-associated pathways in malignant and non-malignant cells of 24 PAAD samples. (B) The expression levels of different URGs in malignant and non-malignant cells of 24 PAAD samples. (PNG 1164 kb) [file 10142_2023_1158_Fig14_ESM.png]

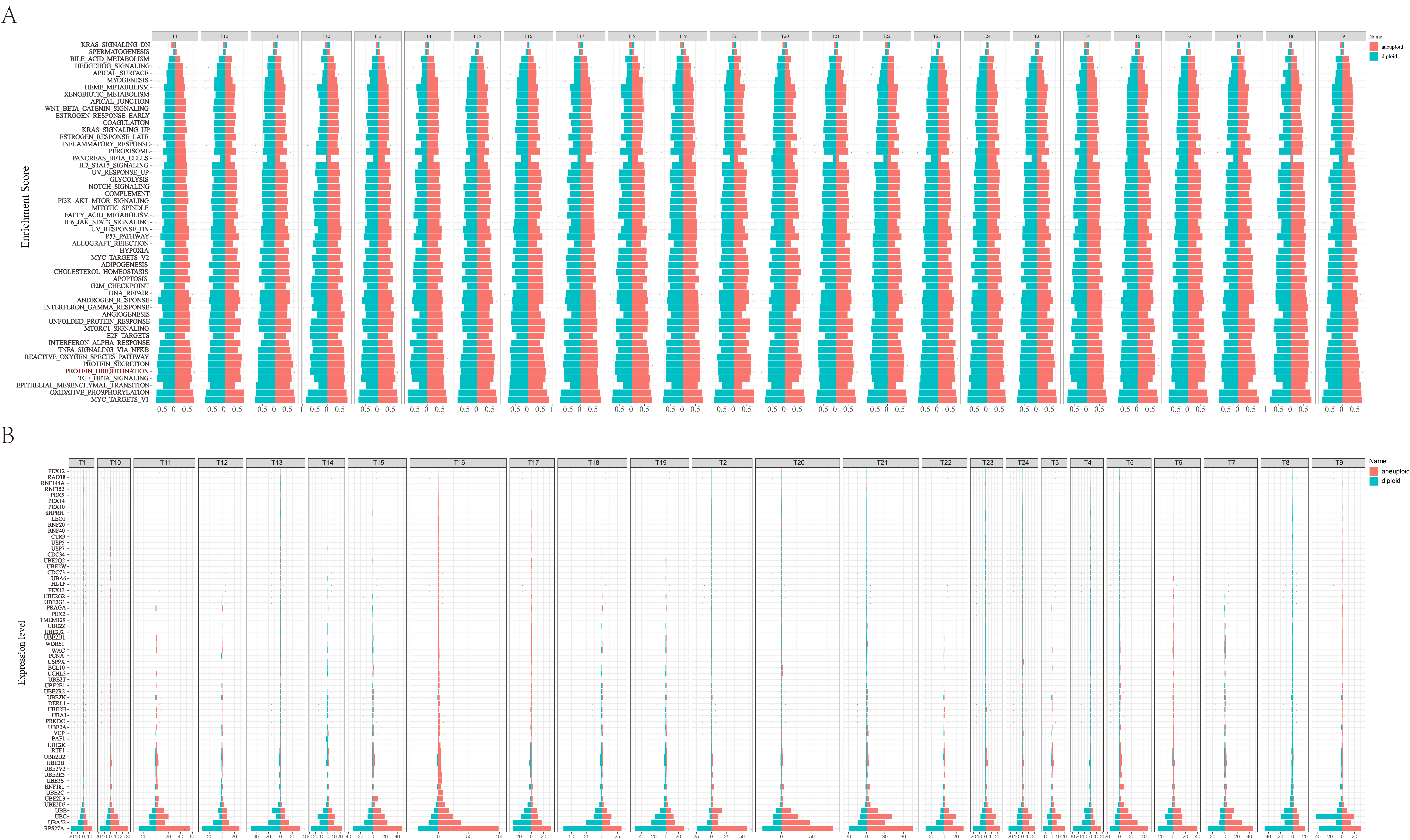

Supplement: Supplementary file 9 — High resulotion image (TIF 17582 kb) [file 10142_2023_1158_MOESM7_ESM.tif]

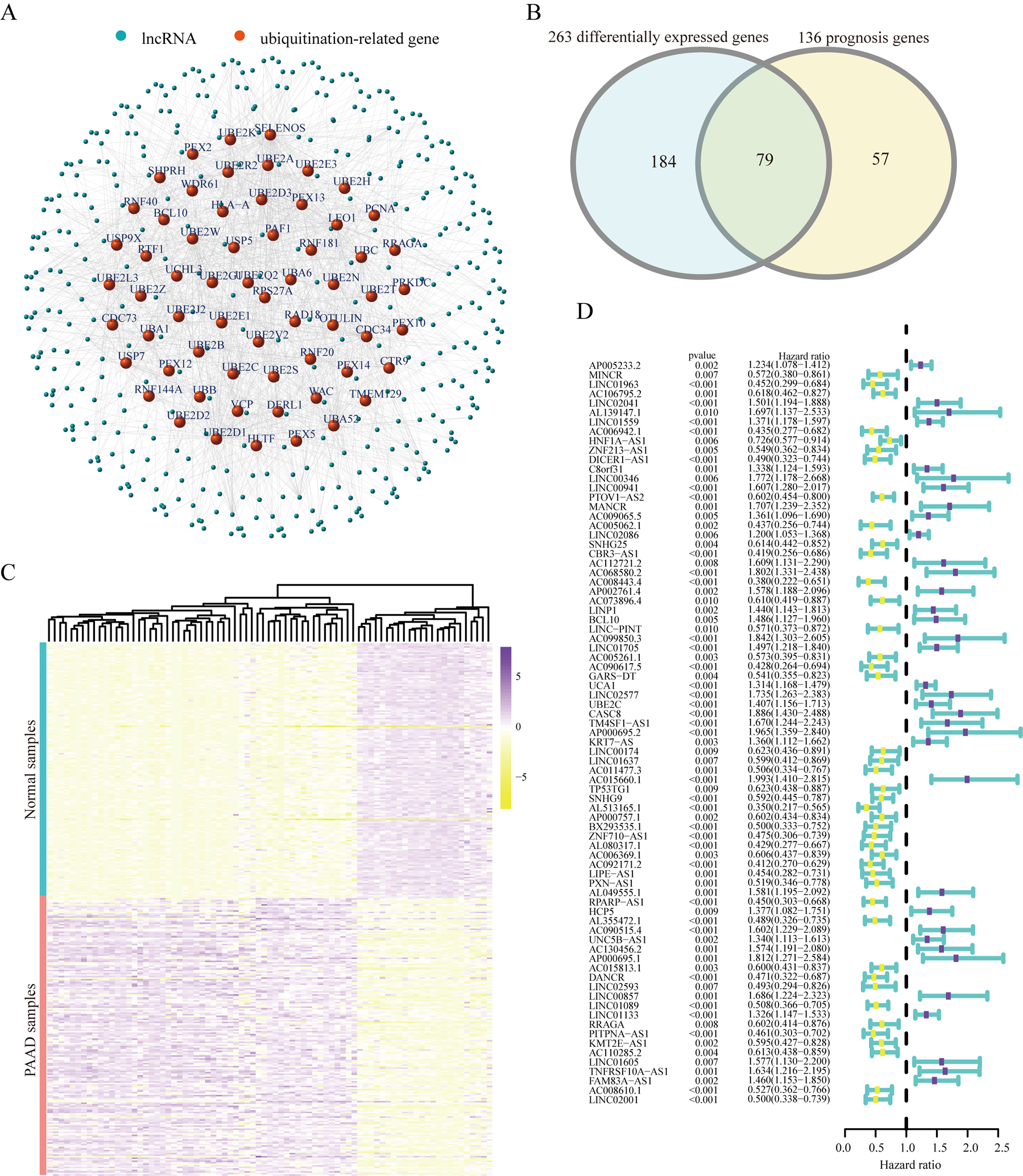

Supplement: Supplementary file 10 — Supplementary Figure 3 Construction of mRNA-lncRNA co-expressed network and scanning of specific ubiquitination-related mRNA and lncRNA with prognostic values. (A) Construction of mRNA-lncRNA co-expressed network. Pearson correlation analysis showed the correlation between 64 ubiquitination-related mRNAs and 10119 lncRNAs (|R| >0.4 and P<0.01). (B) Identification of differentially expressed mRNAs and lncRNAs with prognostic values. (C) The heatmap displayed the expression distributions of specific mRNAs and lncRNAs in PAAD and normal samples. (D) The forest plot displayed the prognostic performances of specific mRNAs and lncRNAs in PAAD. (PNG 2064 kb) [file 10142_2023_1158_Fig15_ESM.png]

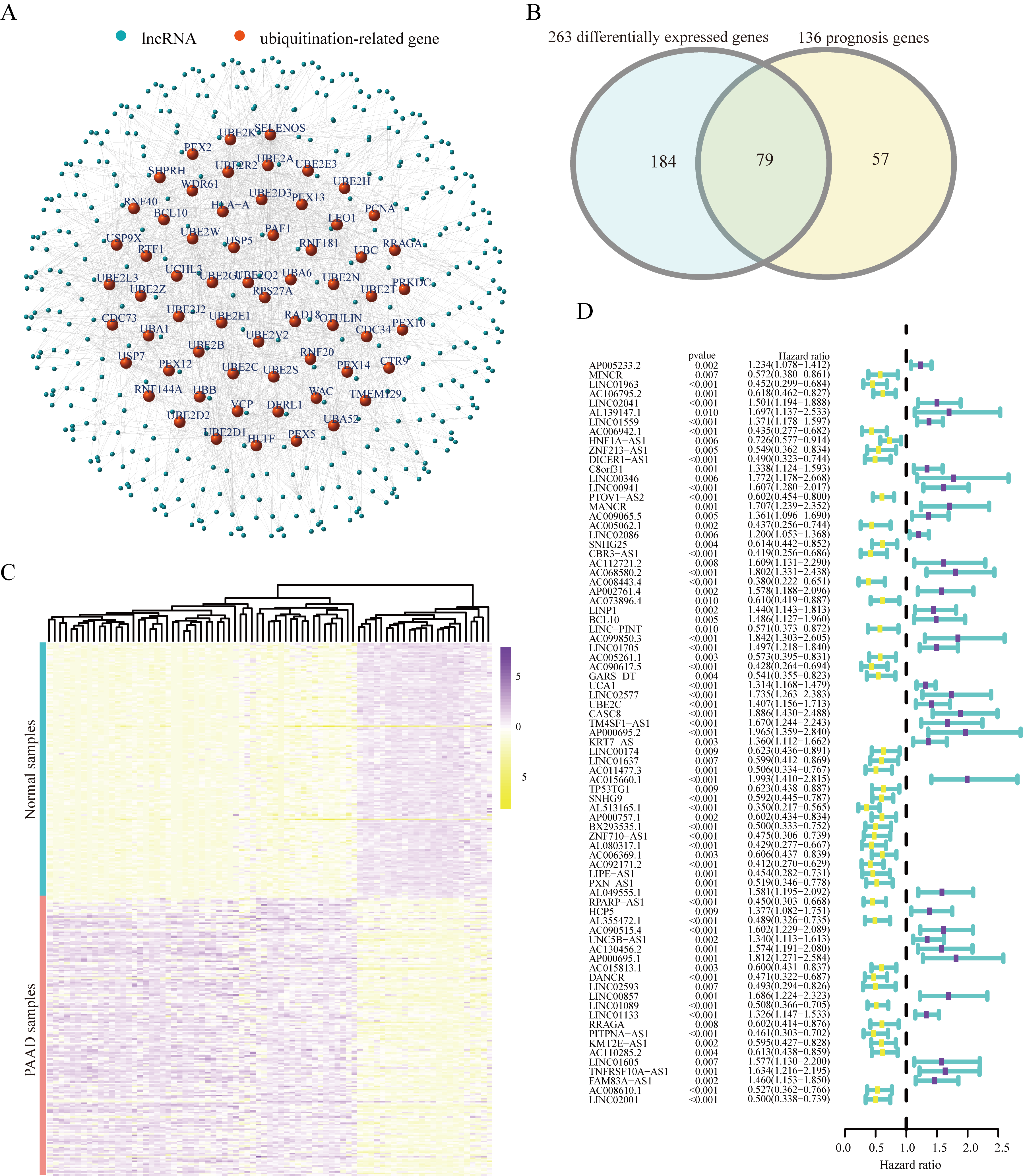

Supplement: Supplementary file 11 — High resulotion image (TIF 10574 kb) [file 10142_2023_1158_MOESM8_ESM.tif]

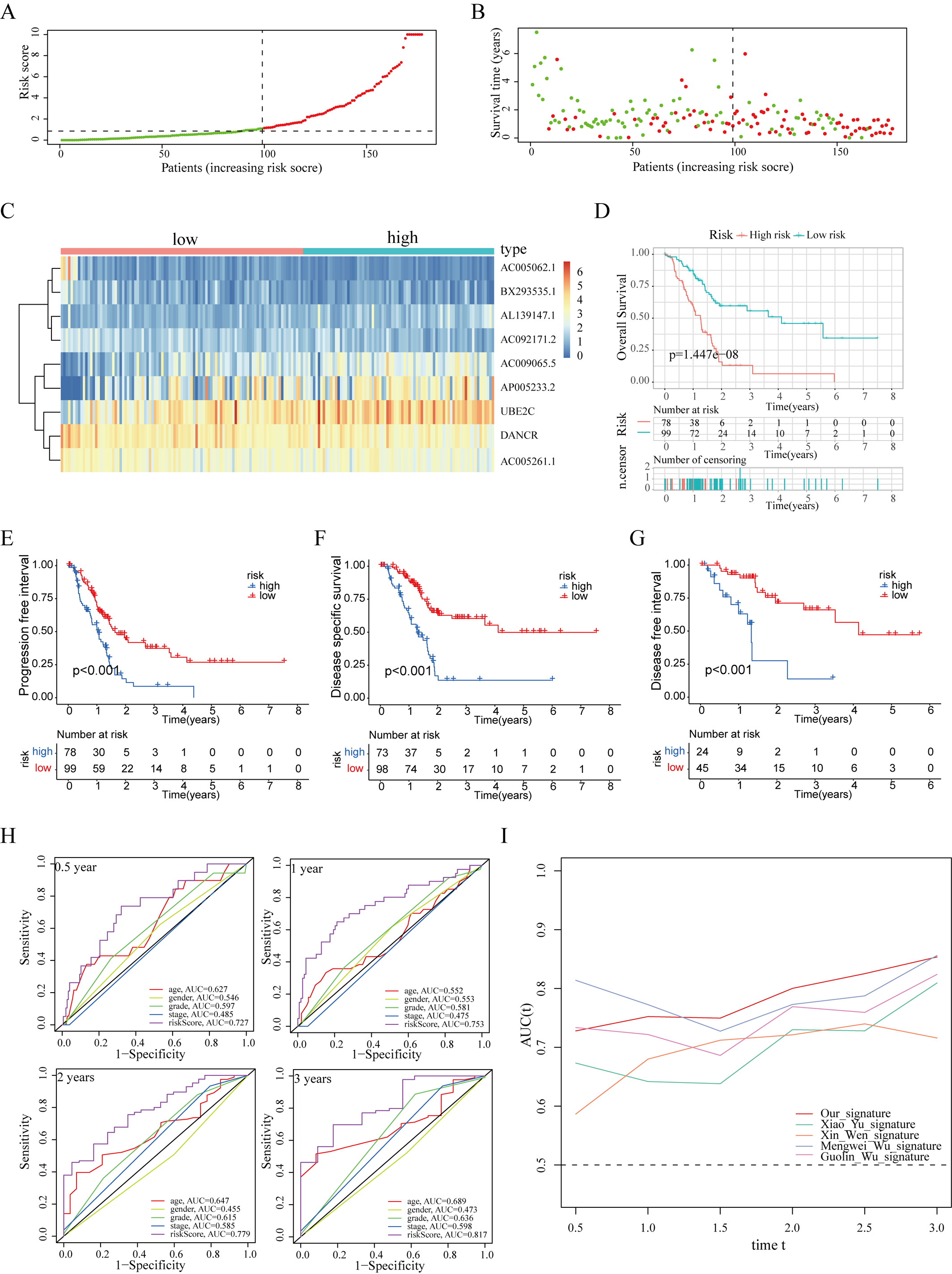

Supplement: Supplementary file 12 — Supplementary Figure 4 The internal dataset of test1 cohort verifies the effectiveness of the ubiquitination-related prognostic panel. (A) Sectionalization of different risk subpopulations in the test1 cohort. (B) Correlation between risk score and clinical outcomes in the test1 cohort. (C) The distributions of panel genes in different risk subpopulations in the test1 cohort. (D-G) Survival analysis of OS, PFI, DSS, and DFI in different risk subpopulations. (H) ROC curves showed the AUC values of clinical features and risk scores in the test1 cohort. (I) ROC curves of our URPS and other four prognostic signatures of PAAD. (PNG 991 kb) [file 10142_2023_1158_Fig16_ESM.png]

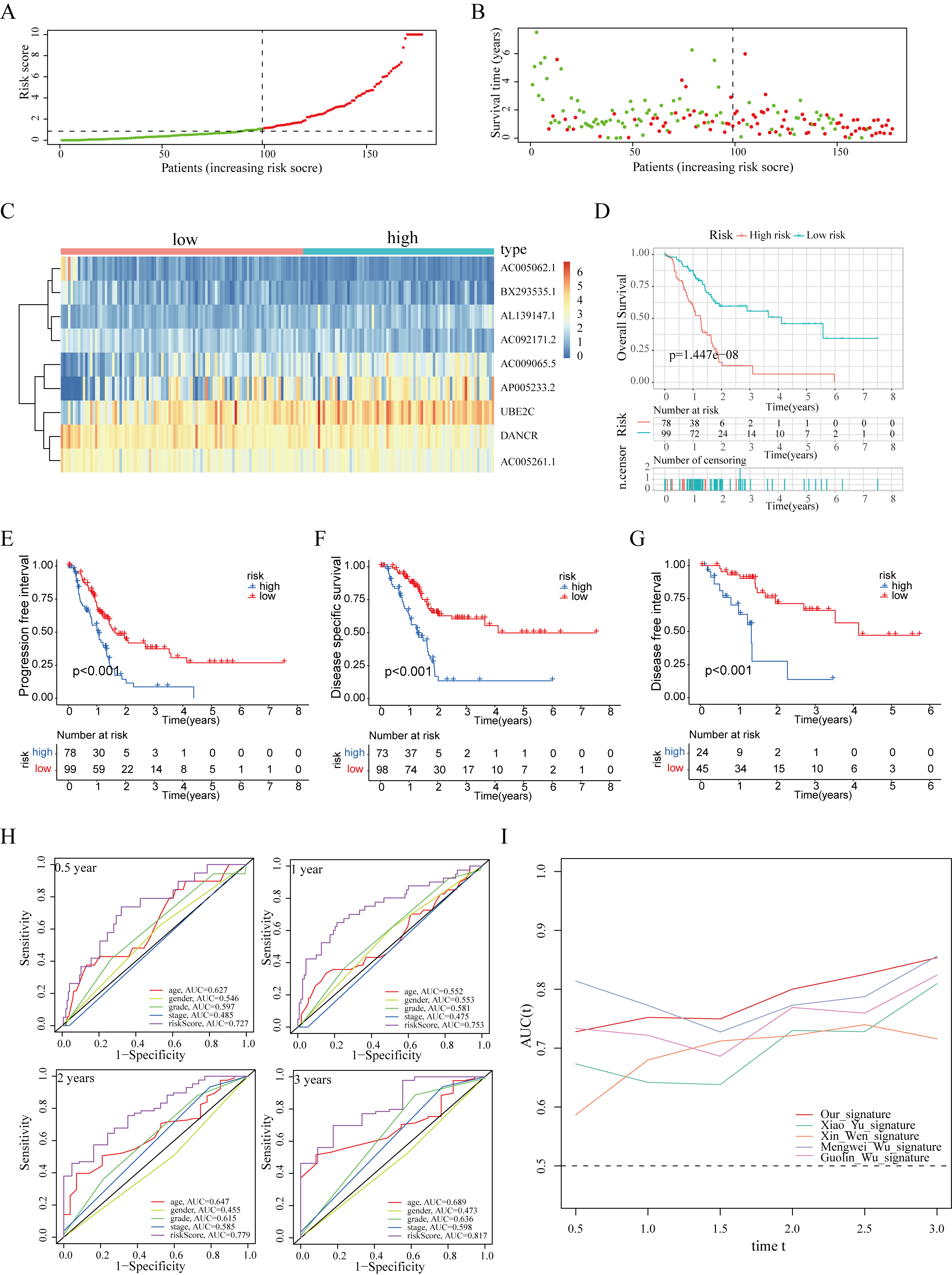

Supplement: Supplementary file 13 — High resulotion image (TIF 7071 kb) [file 10142_2023_1158_MOESM9_ESM.tif]

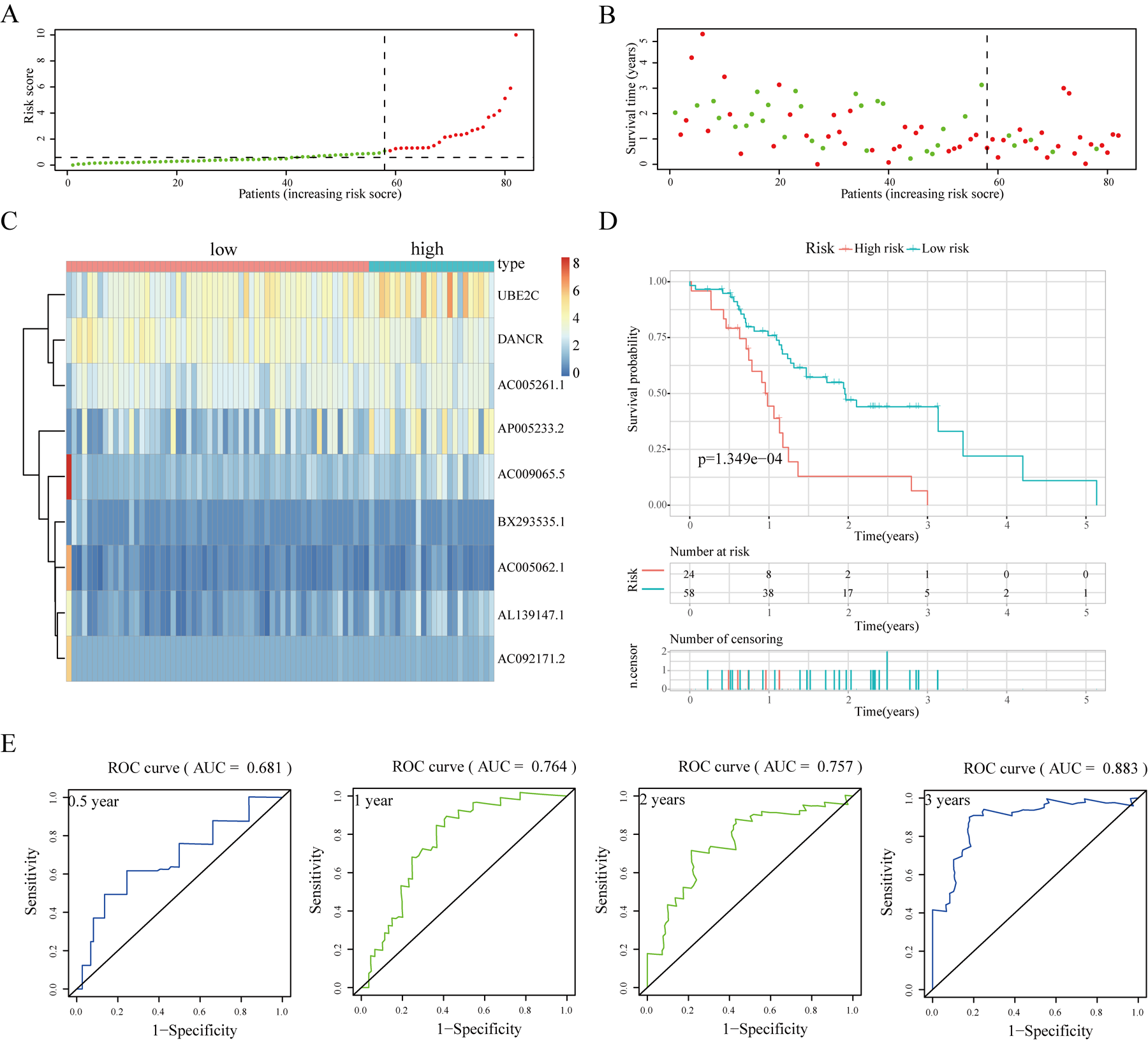

Supplement: Supplementary file 14 — Supplementary Figure 5 The external dataset of test2 cohort verifies the robustness of the ubiquitination-related prognostic signature. (A) Sectionalization of different risk subpopulations in the test2 cohort. (B) Correlation between risk score and clinical outcomes in the test2 cohort. (C) The distributions of panel genes in different risk subpopulations in the test2 cohort. (D) Survival analysis of OS in different risk subpopulations. (E) ROC curves showed the AUC values of clinical features and risk score in the test2 cohort. (PNG 352 kb) [file 10142_2023_1158_Fig17_ESM.png]

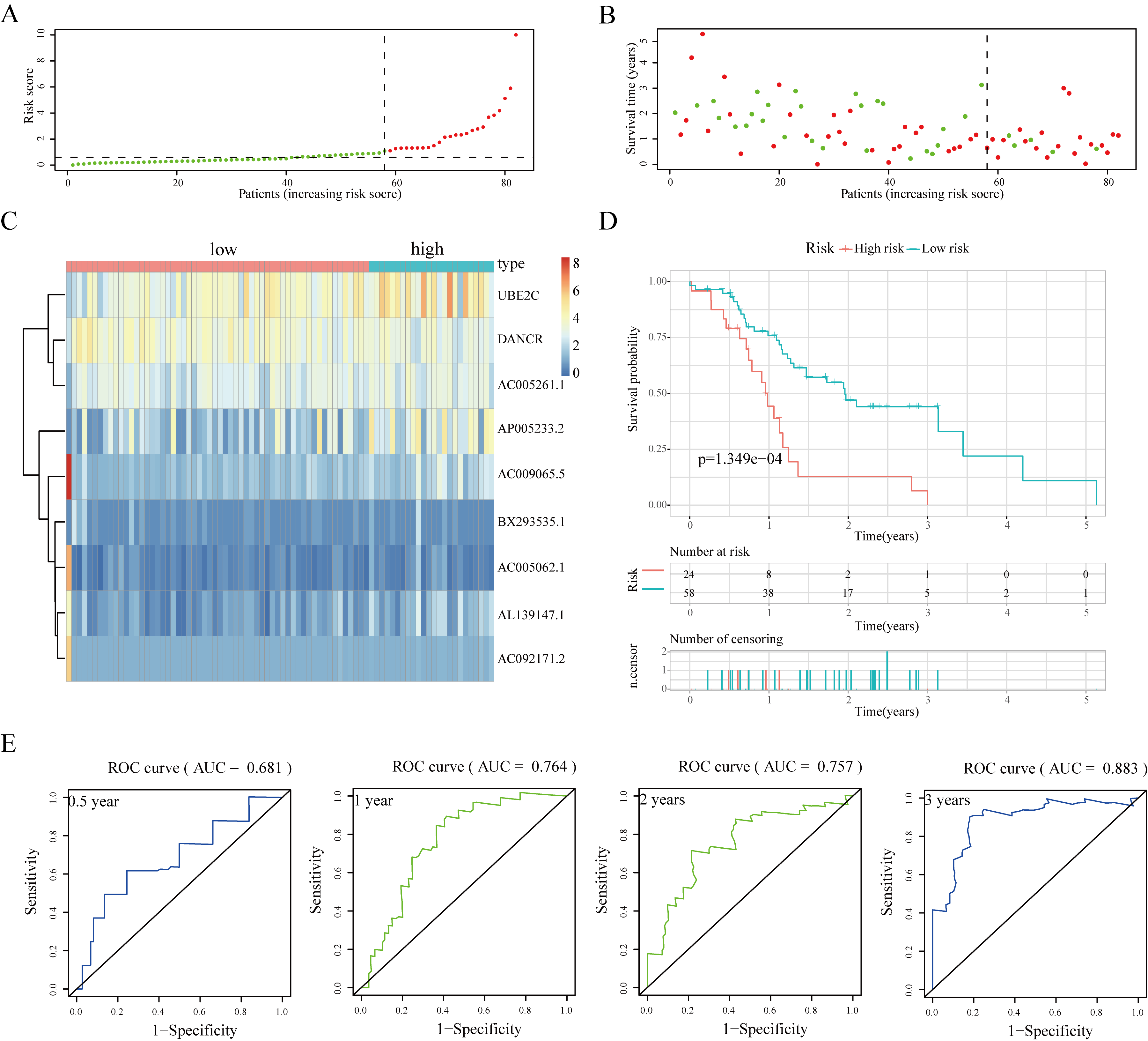

Supplement: Supplementary file 15 — High resulotion image (TIF 4208 kb) [file 10142_2023_1158_MOESM10_ESM.tif]
